# Chromatin accessibility landscapes of immune cells in rheumatoid arthritis nominate monocytes in disease pathogenesis

**DOI:** 10.1186/s12915-021-01011-6

**Published:** 2021-04-16

**Authors:** Dandan Zong, Beibei Huang, Young Li, Yichen Lu, Nan Xiang, Chuang Guo, Qian Liu, Qing Sha, Pengcheng Du, Qiaoni Yu, Wen Zhang, Pengfei Cai, Yanping Sun, Jinhui Tao, Xiaomei Li, Shanbao Cai, Kun Qu

**Affiliations:** 1 Department of Rheumatology and Immunology, The First Affiliated Hospital of USTC, Division of Molecular Medicine, Hefei National Laboratory for Physical Sciences at Microscale, Division of Life Sciences and Medicine, University of Science and Technology of China, Hefei, 230021 Anhui China; 2 Department of Rheumatology and Immunology, The First Affiliated Hospital of USTC, Division of Life Sciences and Medicine, University of Science and Technology of China, Hefei, 230021 Anhui China; 3 Department of Orthopaedics and Bone Oncology, The First Affiliated Hospital of USTC, Division of Life Sciences and Medicine, University of Science and Technology of China, Hefei, Anhui, 230021 China; 4 CAS Center for Excellence in Molecular Cell Sciences, the CAS Key Laboratory of Innate Immunity and Chronic Disease, University of Science and Technology of China, Hefei, 230027 Anhui China; 5 School of Data Science, University of Science and Technology of China, Hefei, 230027 China

**Keywords:** Rheumatoid arthritis (RA), Chromatin dysregulation, Monocytes, C-reactive protein, FRA2

## Abstract

**Background:**

Rheumatoid arthritis (RA) is a chronic, systemic autoimmune disease that involves a variety of cell types. However, how the epigenetic dysregulations of peripheral immune cells contribute to the pathogenesis of RA still remains largely unclear.

**Results:**

Here, we analysed the genome-wide active DNA regulatory elements of four major immune cells, namely monocytes, B cells, CD4^+^ T cells and CD8^+^ T cells, in peripheral blood of RA patients, osteoarthritis (OA) patients and healthy donors using Assay of Transposase Accessible Chromatin with sequencing (ATAC-seq). We found a strong RA-associated chromatin dysregulation signature in monocytes, but no other examined cell types. Moreover, we found that serum C-reactive protein (CRP) can induce the RA-associated chromatin dysregulation in monocytes via in vitro experiments. And the extent of this dysregulation was regulated through the transcription factor FRA2.

**Conclusions:**

Together, our study revealed a CRP-induced pathogenic chromatin dysregulation signature in monocytes from RA patients and predicted the responsible signalling pathway as potential therapeutic targets for the disease.

**Supplementary Information:**

The online version contains supplementary material available at 10.1186/s12915-021-01011-6.

## Background

Rheumatoid arthritis (RA) is a chronic autoimmune disease that affects about 0.5–1% of the population [1]. Patients with severe RA can suffer from irreversible disability and an inability to work, and the mortality rate of RA patients is higher than in healthy people. The pathology of RA is not fully understood, but aberrant immune system function is believed to be essential for RA pathogenesis [2]. Infiltration of T cells, B cells, and monocytes in the synovial membranes enhances the inflammation environment and cartilage damage in RA [3]. However, cartilage damage is also observed in other degenerative joint disorders such as osteoarthritis (OA) [4]. Despite the mechanistic and phenotypic differences between RA and OA, cartilage damage is understood as a key trigger of inflammation at the joints in both diseases [4]. Therefore, OA is often used as control in researches of RA to reveal the autoimmune characteristics of RA.

Inflammatory cytokines play essential roles in the pathogenesis of RA. For example, tumour necrosis factor (TNF)-α, interleukin (IL)-1β, and IL-6 enhance osteoclastogenesis at the joints, which leads to bone erosion [5]. Elevation of TNF, IL-1β, and IL-6 induces the production of CRP [6, 7], another inflammatory factor that reflects disease activity of RA [8, 9] .CRP functions in a variety of ways to promote RA pathogenesis. For instance, CRP binds to Fcγ receptors and mediates the secretion of inflammatory cytokines to initiate proinflammation reactions [10]. Besides, CRP triggers bone destruction by activating RANKL to promote osteoclastogenesis in RA [11]. Thus, biologic drugs targeting inflammatory pathways, such as TNF-α and IL-6 signals, have been recommended to treat RA in combination with conventional synthetic drugs, and such combinations confer significantly better clinical efficacy than synthetic drugs alone [12].

In addition to dysregulation at genetic level, RA patients also exhibit epigenetic abnormalities [13]. The DNA methylation and histone modification of the immune cells in the joints are generally altered in RA, and such modifications contribute to activation of immune cells [14]. Besides local impacts in joints, many RA-related features are present in the peripheral blood of patients, such as the appearance of autoantibodies and CRP, elevated inflammatory cytokines [3], which affect other parts of the body through the circulation system. Thus, RA-related epigenetic dysregulation is also observable in peripheral samples. DNA methylation alteration is the most common epigenetic dysregulation in peripheral immune cells, including a global DNA hypomethylation in T cells and monocytes [15]. However, it remains unclear how peripheral epigenetic dysregulation is induced and how such dysregulation contributes to RA pathogenesis.

Here, we examined the chromatin states of four main immune cell types in the peripheral blood from RA patients using Assay of Transposase Accessible Chromatin with sequencing (ATAC-seq) [16]. We detected RA-associated chromatin dysregulation in peripheral monocytes that promote the RA pathogenesis and found that serum CRP of RA patients is responsible for it.

## Results

### Immune cells in peripheral blood exhibit extensive RA-associated chromatin dysregulation

**Fig. 1:**
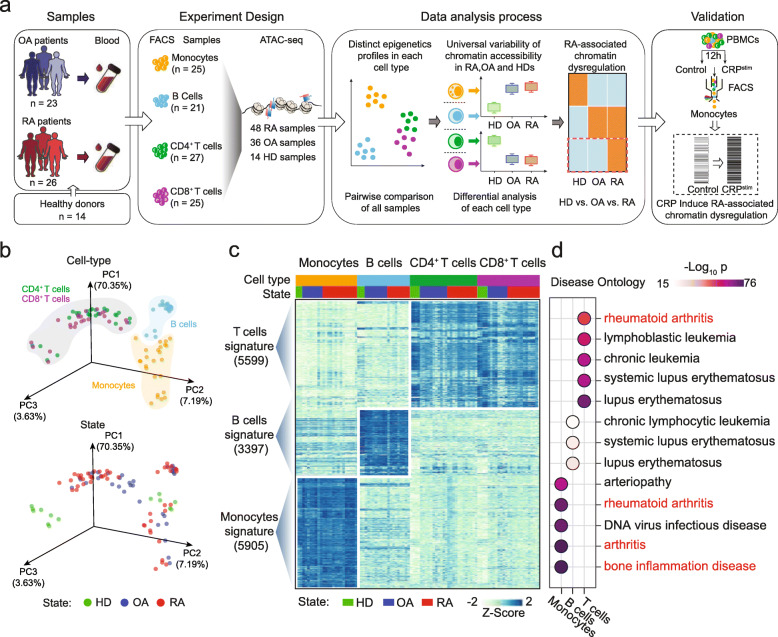
Chromatin accessibilities of major immune cell types in PBMCs from OA, RA, and HD. **a** Schematic outline of the study design, depicting the workflow for ATAC-seq library construction of the immune cells from peripheral blood of OA patients, RA patients, and healthy donors, as well as the data analysis and experimental verification steps. **b** Principal component analysis based all chromatin accessible regions for all samples. Each dot is a sample, the samples in the figure are colour-coded by cell-type (top) and state (bottom). **c** Using a screening condition of FDR < 0.01, | log_2_ fold change | > 4 and *p* < 0.001 for a pairwise comparison between all samples, the heatmap shows the peak intensity of significantly different peaks after unsupervised clustering. Colour bars show the cell-type and state of samples. Unsupervised clustering of differential peaks is performed using K-means algorithm with the k value of 3. **d** Representation of selected disease ontology categories obtained from the analysis of cell type-specific (identified in Fig. 1c) chromatin accessible regions using the Genomic Regions Enrichment of Annotations Tool (GREAT). HD, healthy donor; OA, osteoarthritis; RA, rheumatoid arthritis

We collected peripheral blood samples directly from 26 RA patients and 23 age- and sex-matched OA patients (Table 1, Additional file 2: Table S1). We sorted monocytes, B cells, CD4^+^ T cells, and CD8^+^ T cells (Additional file 1: Figure S1a, b) and then constructed ATAC-seq libraries for high throughput sequencing. Additionally, we downloaded 14 ATAC-seq profiles for the same immune cell types from peripheral blood of healthy donors from the GEO database (GSE118189 [17], GSE74912 [18]) as healthy controls (Fig. 1a). We used a published ATAC-seq pipeline [19] to identify focal peaks for accessible chromatin regions (accessible regions; Fig. 1a). After filtering and quantile normalization, we identified a total of 169,267 high-quality and reproducible accessible regions across these 4 immune cell types (Additional file 1: Figure S1c-e, Additional file 3: Table S2).

**Table 1:** Clinical data of patients included in this study

	Blood samples for ATAC-seq libraries		Blood samples for FACS analysis		Blood samples for CRP stimulation
	RA patients	OA patients	RA patients	OA patients	OA patients
Number of patients	26	23	8	5	8
Gender, F/M	21/5	19/4	8/0	3/2	4/4
Age (years)	54 (48–66)	61 (54–67)	54.5 (49.5–68.75)	61 (57–62)	65 (62–67.75)
RF positive (%)	76.9	0	75	0	0
Anti-CCP positive (%)	73.1	NA	87.5	NA	NA
TCJ	10 (3–12)	NA	11 (4.75–14)	NA	NA
SCJ	10 (4–12)	NA	10 (3.5–11.5)	NA	NA
GH	70 (50–80)	NA	75 (50–80)	NA	NA
ESR	30 (16–42)	11 (10–14.75)	29.5 (15.75–46.75)	14 (8–19)	8 (6–18.5)
CRP	22.9 (6.8–44.3)	3.13 (3.13–3.14)	30.22 (6.75–53.95)	3.11 (3.11–3.12)	3.055 (2.97–7.3)
DAS28-ESR	6 (5–6)	NA	6 (4.75–7)	NA	NA
DAS28-CRP	6 (5–6)	NA	6 (5.25–6)	NA	NA

Values provide the median with IQR

*F/M* female/male, *ESR* erythrocyte sedimentation rate, *CRP* C-reactive protein, *RF* rheumatoid factor, *Anti-CCP* anti-cyclic citrullinated peptide, *TCJ* tender joint count, *SCJ* swollen joint count, *GH* patient assessment of disease activity using a 100-mm visual analogue scale (VAS) with 0 = best and 100 = worst, *DAS28-ESR* Disease Activity Score 28-erythrocyte sedimentation rate, *DAS28-CRP* Disease Activity Score 28-C-reactive protein, *FACS* flow cytometry, *NA* not account

Next, we performed a principal component analysis (PCA) for all samples, which indicated that disease-specific differences in accessible regions were overwhelmed by cell-type-specific differences (Fig. 1b). We also performed a pairwise comparison of the ATAC-seq profiles between each of the four immune cell types, which again emphasized that the differences inherent to the cell types overwhelmed the disease-specific trends in chromatin states (Fig. 1c). These results clearly revealed the necessity to inspect the different cell types individually. Pursuing this, we used GREAT [20] annotations to explore the disease ontology categories of cell-type-specific accessible regions, which indicated that “rheumatoid arthritis”-associated genomic loci were highly enriched among the monocyte- and T cell-specific accessible regions (Fig. 1d). Thus, we hypothesized that the altered accessible regions of peripheral immune cells in RA patients reflect the RA-associated chromatin dysregulation, which may contribute to the pathogenesis of RA.

### RA-associated chromatin dysregulation in peripheral monocytes represents an RA signature

**Fig. 2:**
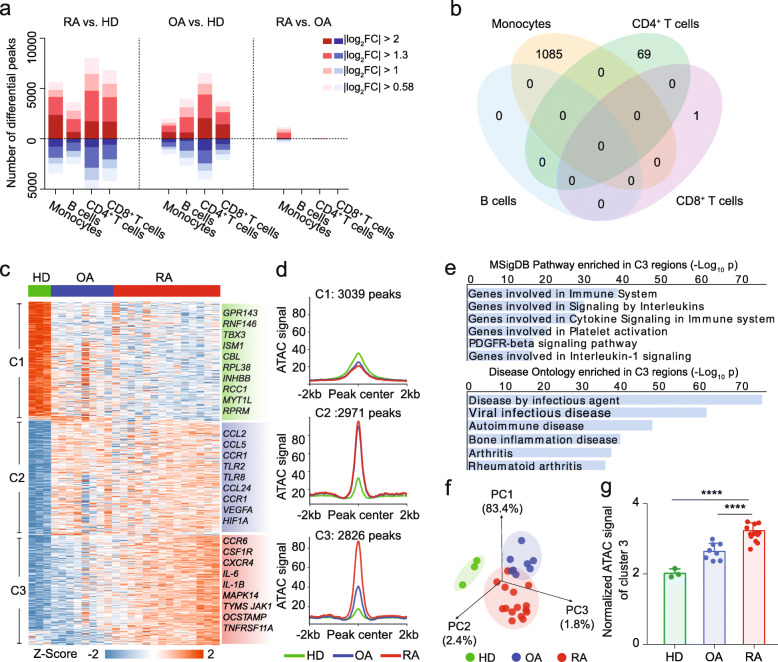
RA-associated chromatin dysregulation in peripheral monocytes represents an RA signature. **a** Barplot showing the numbers differentially accessible chromatin regions for each of the four examined immune cell types, binned according to the magnitude of the detected OA and RA vs. HD, coloured according to the fold change. RA vs. HD (left), OA vs. HD (middle), and RA vs. OA (right). **b** Venn diagram for the overlap of differentially accessible chromatin regions in the four examined immune cell types from the RA vs. OA analysis (|log_2_ fold change | > 1, *p* < 0.001 and FDR < 0.1). **c** Heatmap of 8836 peaks obtained by comparing the ATAC-seq profiles of monocytes from healthy donors, OA patients, and RA patients (|log_2_ fold change | > 1, *p* < 0.001 and FDR < 0.1). Each column is a sample; each row is an accessible region detected by ATAC-seq; peaks are organized based on unsupervised clustering. Samples from the same group are marked with the same colour. Unsupervised clustering of differential peaks is performed using K-means algorithm with the *k* value of 3. **d** Diagram for the average peak intensity signals of three clusters (C1, C2, and C3) for healthy donors, OA patients, and RA patients. **e** GREAT was used to analyse C3 peaks for enrichment of disease ontology categories and MsigDB pathways. **f** Principal component analysis based on the C3 peaks for all samples. Each dot is a sample. **g** Barplot of normalized signal intensity of C3 peaks (RAAS) in healthy donors, OA patients and RA patients. The values of *p* were calculated with an unpaired *t*-test. ****p < 0.0001. Error bar represent standard deviation (SD). HD, healthy donor; OA, osteoarthritis; RA, rheumatoid arthritis; C1, cluster 1; C2, cluster 2; C3, cluster 3

We next examined the chromatin states of the different peripheral immune cell types among RA patients, OA patients, and HD in detail. Compared with HD, there were thousands of significant differentially accessible regions in the OA and RA patients for all examined cell types (Fig. 2a). However, when we compared RA with OA, we found 1085, 69, 1, and 0 differentially accessible regions in monocytes, CD4^+^ T cells, CD8^+^ T cells, and B cells, respectively (|log_2_ fold change | > 1, *p* < 0.001, FDR < 0.1), and the differentially accessible regions between cell types exhibited barely any overlap (Fig. 2a, b). Since the distributions of monocyte subtypes may differ among patients, we used surface expression of the CD14 and CD16 to catalogue the monocyte subpopulations in peripheral blood: there were no significant differences for monocyte subtype distributions between RA and OA patients (Additional file 1: Figure S2a). This result revealed that the chromatin dysregulation in monocytes of RA patients may result from general changes in chromatin status, rather than an imbalance in the distribution of monocyte subtypes.

To further investigate the characteristics of the detected differentially accessible regions in monocytes among RA patients, OA patients, and HD, we performed pairwise comparison and unsupervised clustering analyses, which grouped all the differentially accessible regions into three distinct clusters (|log_2_ fold change | > 1, *p* < 0.001, FDR < 0.1; C1, C2, C3; Fig. 2c; Additional file 4: Table S3). C1 regions were more accessible in HD, representing a normal chromatin state signature. C2 regions were more accessible in both OA and RA patients, representing a disease signature. C3 regions were more accessible in RA compared to OA and to HD, representing an RA signature (Fig. 2c, d). Notably, similar analyses of the chromatin states in the other examined cell types revealed clusters of more or less accessible regions in both OA and RA patients, but no obvious clusters specific to RA (Additional file 1: Figure S2b).

By annotating functions of C1–C3 regions with GREAT, we observed no significant enrichment of particular disease ontologies or biological functions for C1 regions (Additional file 1: Figure S2c). C2 regions were highly enriched for cytokine and immune system-associated signalling pathways and autoimmune disease ontology (Additional file 1: Figure S2c), consistent with the known functional impacts of cytokines and immune response in both RA and OA [4]. C3 regions were also strongly enriched for cytokine, immune-associated pathways and autoimmune disease ontology. In addition, “bone inflammation disease” and “rheumatoid arthritis” ontologies were highly enriched in C3 regions (Fig. 2e), confirming that C3 regions represent an RA signature, for example, *IL-1B* [21] and *JAK1* [22], which are essential for proinflammatory signal transduction and upregulated in RA patients (Additional file 1: Figure S3a). Moreover, we applied a widely used analysis tool named ChromHMM [23] to characterize the functional genomic features of C3 peaks. We found that these peaks were highly enriched in promoters and active enhancers (Additional file 1: Figure S3b). Besides, we clustered the differential peaks of B and T cells with the same parameters and found no enriched RA-associated disease ontologies in them (Additional file 1: Figure S2b, c). To further confirm that C3 regions represent an RA signature, we performed a PCA analysis with C1, C2, and C3 regions for RA patients, OA patients, and HD. Only when C3 regions were used as features, the PCA model separated RA patients from OA patients and from HD obviously (Fig. 2f); the models for the C1 and C2 regions both failed to separate out the RA patients (Additional file 1: Figure S4). Moreover, we calculated the normalized signal intensity of C3 regions as a C3 score for each patient monocyte sample (see the “Methods” section). Unsurprisingly, the C3 scores of RA patients were significantly higher than those for OA patients or HD (Fig. 2g). Thus, we defined this normalized signal intensity of C3 regions as “RA-associated ATAC-seq score” (RAAS), which meaningfully represents the extent of RA-associated chromatin dysregulation in patients.

### RA-associated chromatin dysregulation is correlated with the serum CRP levels of RA patients

**Fig. 3:**
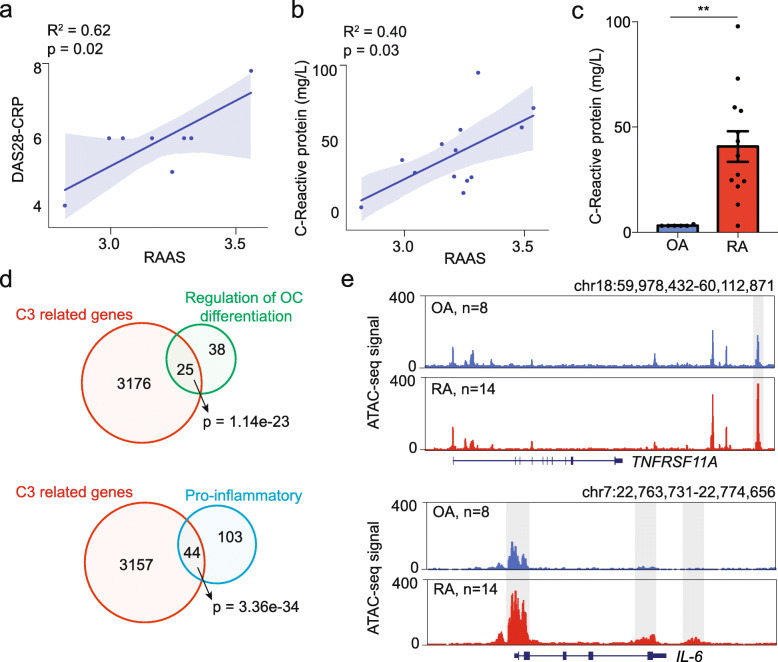
RA-associated chromatin dysregulation is positively correlated with the serum CRP levels of RA patients. **a**, **b** Linear regression analysis was used to compare RAAS with DAS28-CRP score (**a**) and with CRP serum concentration (**b**). The shading represents the confidence interval. The solid line was fit from linear regression, and the *p* value and the square of the coefficient of correlation (*R*^2^) were calculated using the “OLS” function in the statsmodels package in Python. *P* < 0.05 were considered significant. **c** Barplot showing the average serum CRP levels in OA and RA patients. The values of *p* were calculated with an unpaired *t*-test. **p < 0.01. Error bar represent standard error of the mean (SEM). **d** Venn diagrams showing the numbers and *p* for overlap between the regulation of OC differentiation gene set or the proinflammatory gene set and C3-related genes (identified by GREAT). *P* was calculated using Fisher’ exact test, ****p < 0.0001. **e** Normalized ATAC-seq profiles at the TNFRSF11A and IL-6 loci in OA and RA patients. Shaded regions are more accessible representative peaks in RA. OC, osteoclast; OA, osteoarthritis; RA, rheumatoid arthritis; C3, cluster 3; DAS28-CRP: disease activity score DAS28 based on C-reaction protein levels; RAAS, RA-associated ATAC-seq score

To explore whether the RA-associated chromatin dysregulation we detected has clinical significance, we then performed a Pearson correlation analysis between the RAAS and the disease activity score 28 (DAS28) for the RA patients, which is a widely utilized assessment for RA clinical disease activity [12]. There are different versions of DAS28, including DAS28-CRP (based on the serum CRP level) and DAS28-ESR (based on the ESR, erythrocyte sedimentation rate) [24]. We detected a significant positive correlation of RAAS with DAS28-CRP but not DAS28-ESR (Fig. 3a, Additional file 1: Figure S5a), suggesting that the RAAS is associated with RA disease activity and CRP levels may also correlate with the RA-associated chromatin dysregulation. Therefore, we examined whether there were any correlations between values of RAAS with CRP levels and other clinical indicators of RA (TCJ, SCJ, RF, ESR, and ACPA), which showed that only CRP had a significant correlation with RAAS (Fig. 3b, Additional file 1: Figure S5b). Consistent with previous reports [25, 26], we also found that the RA patients had significantly higher serum CRP levels compared to the OA patients (Fig. 3c).

Previous studies have shown that serum CRP stimulation promotes differentiation of osteoclast (OC) and expression of proinflammatory genes in monocytes [11, 27]. So we speculated that the correlation between RAAS and CRP indicate some involvement of RA-associated chromatin dysregulation in OC differentiation and/or proinflammatory bioprocesses. Pursuing this, we annotated C3 region-related genes with GREAT and tested the enrichment for gene sets of “regulation of OC differentiation” and “proinflammation” (from MSigDB [28]) in C3 region-related genes (Additional file 5: Table S4). We found that both gene sets were highly enriched in C3 (Fig. 3d). For example, the genomic locus around *TNFRSF11A* (RANK), the receptor of the RANKL signal that is known to be induced by serum CRP [11] to promote OC differentiation [29], was preferentially more accessible in RA patients compared to OA patients. The chromatin state around *IL-6*, a gene known to be induced by CRP that promotes the inflammatory pathogenesis of RA [27], was also in a more accessible chromatin state in RA patients than in OA patients (Fig. 3e). These findings suggest that high serum CRP of RA patients may induce RA-associated chromatin dysregulation in monocytes.

### CRP stimulation induced RA-associated chromatin dysregulation in monocytes

**Fig. 4:**
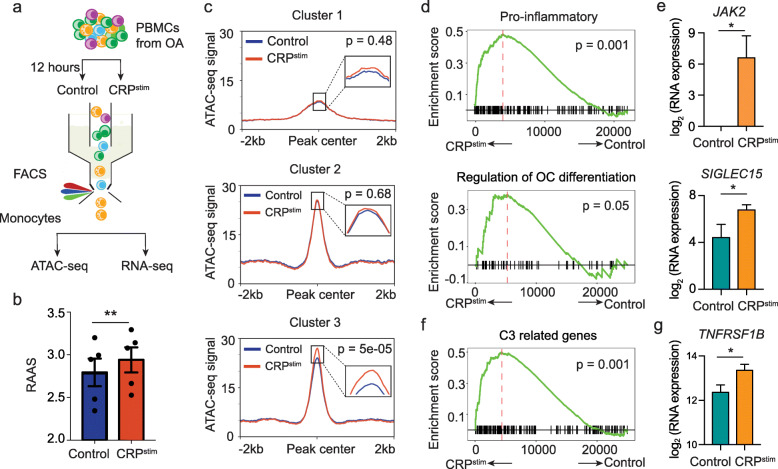
CRP stimulation induced RA-associated chromatin dysregulation in monocytes. **a** Schematic depicting the experimental design for CRP stimulation in vitro. PBMCs of OA patients were co-cultured for 12 h with or without CRP, and then monocytes were sorted and used to generate ATAC-seq and RNAseq libraries. **b** Barplot showing RAASs before and after CRP stimulation in monocytes. The *p* value was estimated from paired Student’s *t* test. **p < 0.01. Error bar represent standard error of the mean (SEM). **c** Diagrams for the average ATAC-seq signal intensity for C1-C3 peaks of control and CRP stimulation in monocyte. The values of *p* were calculated for the comparison of the average signals within 10 bp of the peak centres using unpaired *t*-test. **d**, **f** GSEA enrichment plots highlighting RNA-seq signals for proinflammation (**d**), regulation of OC-differentiation (**d**), and genes related to RA-associated dysregulation (**f**) of the running enrichment scores (ES) and positions of gene set members among the rank-ordered list from the GSEA. **e**, **g** The barplot shows the gene expression of *JAK2* (**e**), *SIGLEC15* (**e**), and *TNFRSF1B* (**g**) in the control and CRP stimulation. The *p* value was estimated from unpaired Student’s *t* test. *p < 0.05. CRP, C-reaction protein; OA, osteoarthritis; OC, osteoclast; PBMC, peripheral blood mononuclear cell; CRP^stim^, CRP stimulation; C3, cluster 3; RAAS, RA-associated ATAC-seq score

To investigate whether CRP can induce RA-associated chromatin dysregulation in monocytes, we incubated PBMCs from OA patients with human recombinant CRP or control medium for 12 h in vitro. Then we sorted monocytes to apply ATAC-seq and RNA-seq (Fig. 4a). After filtering and normalization, we obtained reproducible and high-quality ATAC-seq and RNA-seq data of CRP-stimulated and control monocytes (Additional file 1: Figure S6). We first calculated the RAAS values for CRP-stimulated monocytes and observed a significant increase compared to non-stimulated controls (Fig. 4b). Further, we examined the C1–C3 regions in detail and found that only the C3 regions were significantly more accessible in response to CRP stimulation (Fig. 4c). These results establish that CRP stimulation does induce RA-associated chromatin dysregulation. Our RNA-seq analysis showed that CRP stimulation induced expression of myeloid leukocyte activation-related genes in the monocytes from OA patients, according to the GO term analysis of Metasacpe [30] (Additional file 1: Figure S7a). Further GSEA analysis [31] of the RNA-seq data showed that CRP stimulation significantly activated proinflammatory and OC differentiation regulation-related genes in monocytes from OA patients (Fig. 4d, Additional file 1: Figure S7b, c), such as *JAK2* [32] and *SIGLEC15* [33] (Fig. 4e). Moreover, we found that the genes related to C3 regions were strongly enriched in the upregulated genes of CRP-stimulated monocytes, compared to unstimulated controls (Fig. 4f, Additional file 1: Figure S7d), *TNFRSF1B* [34] for instance (Fig. 4g). This results further support that RA-associated chromatin dysregulation is induced by CRP stimulation.

### CRP induces RA-associated chromatin dysregulation via FRA2

**Fig. 5:**
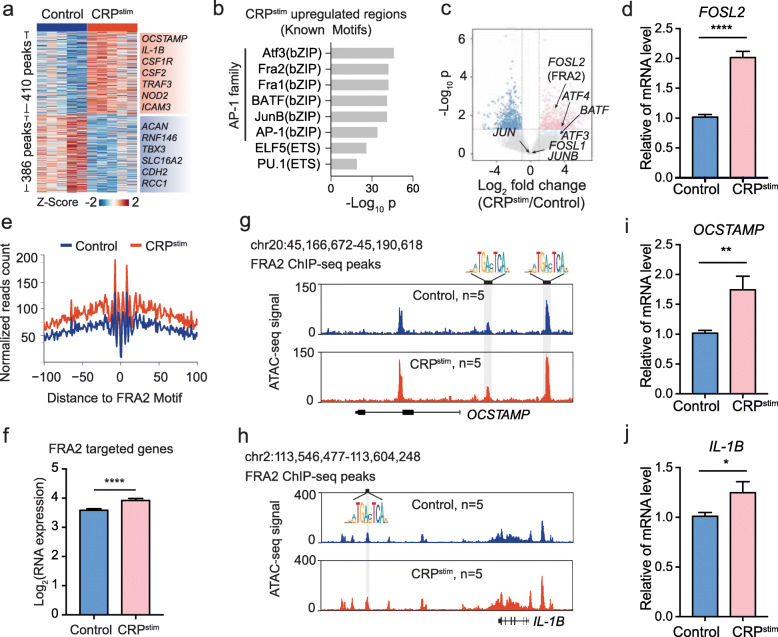
CRP induces RA-associated chromatin dysregulation via FRA2. **a** Heatmap showing changes in chromatin accessibility in monocytes stimulated with the presence or absence of CRP for 12 h (*p* < 0.01 and |fold change| > 1.5). ATAC-seq was used to assess five independent biological replicates. **b** The upregulated peaks of CRP-stimulation were enriched for transcription factors using HOMER and ranked by *p*. **c** Volcano plot showing changes of gene expression in monocytes stimulated with CRP (10 μg/mL) in the presence or absence for 12 h; red dots correspond to upregulated genes with significant (*p* < 0.05 and |fold change| > 1.5). RNA-seq was performed in three independent biological replicates (monocytes derived from three different patients with OA). **d** Expression levels of genes *FOSL2* were confirmed by real-time RT-PCR in control and CRP stimulation group. The *p* value was estimated from unpaired Student’s *t* test. ****p < 0.0001. Error bar represent standard error of the mean (SEM). **e** Visualization of ATAC-seq footprint for FRA2 motifs in control and CRP stimulation. The ATAC-seq signal across all the motif binding sites in the genome were aligned on the motif and averaged. **f** Barplot shows expression level of genes with containing FRA2 binding sites during control and CRP stimulation. Error bar represent standard error of the mean (SEM). The *p* value was estimated from unpaired Student’s t test. ****p < 0.0001. **g**, **h** Normalized ATAC-seq profiles at the *OCSTAMP*(g*)* and *IL-1B*(h) locus in control and CRP^stim^. The shaded regions are more accessible representative peaks containing the FRA2 binding site after CRP stimulation. **i**, **j** Expression levels of genes *OCSTAMP* (**i**) and *IL-1B* (**j**) were confirmed by real-time RT-PCR in control and CRP stimulation group. Error bar represent standard error of the mean (SEM). The *p* value was estimated from unpaired Student’s *t* test. *p < 0.05, **p < 0.01. CRP, C-reaction protein; CRP^stim^, CRP stimulation

CRP has been reported to activate monocytes in RA [11], yet the mechanisms underlying RA-associated chromatin dysregulation in CRP-activated monocytes are poorly understood, and the impacts of any related transcription factors (TFs) remain unknown. We observed 410 significantly more and 386 significantly less accessible chromatin regions after CRP stimulation of OA-patient-derived monocytes (fold change > 1.5, *p* < 0.01; Fig. 5a; Additional file 6: Table S5). Then, we annotated these regions with differential response to CRP, obtained related genes by GREAT, and then performed GO functional enrichment on these genes (see the “Methods” section). We found significant enrichment of GO functional terms such as “leukocyte differentiation” and “osteoclast differentiation” in the peaks that were upregulated after CRP stimulation (Additional file 1: Figure S8a,b). And no immune-associated GO functional terms were enriched in less accessible regions after CRP stimulation (Additional file 1: Figure S8c). This suggested that genes related to upregulated regions are associated with rheumatoid arthritis. Using ChromHMM, we then found that upregulated regions tended to be located in promoters and active enhancers, while downregulated regions were more enriched in quiescent chromatin loci with low transcriptional activity (Additional file 1: Figure S8d). Given that ATAC-seq is able to capture TF binding sites positioned within accessible chromatin regions, we used HOMER [35] to identify any TFs with candidate DNA-binding motifs significantly enriched among the 410 more accessible CRP-responsive regions. Strikingly, 6 of the 8 top-ranked enriched known motifs belonged to TFs of the AP-1 family (Fig. 5b), which was also highly enriched in the RA-associated regions (Cluster 3) of RA patients (Additional file 1: Figure S9). To determine which TF(s) function to conduct the CRP stimulation signal, we examined the RNA expression of AP-1 family TFs in CRP-stimulated monocytes. Compared to untreated controls, we found that only *FOSL2* (FRA2) and *ATF4* were significantly upregulated (fold change > 1.5, *p* < 0.05; Fig. 5c; Additional file 7: Table S6). Viewed alongside our motif enrichment results, we speculated that FRA2 is involved in the CRP-induced RA-associated chromatin dysregulation in monocytes. We then investigated the mRNA expression change of *FOSL2* in response to CRP stimulation and found a significant upregulation (Fig. 5d).

To verify whether FRA2 functions in the CRP-induced RA-associated chromatin dysregulation, we depicted the “footprint” of FRA2 and detected a higher DNA accessibility and a firmer TF occupation flanking FRA2 motifs in CRP-stimulated monocytes compared to unstimulated controls (Fig. 5e), implying a stronger DNA-binding activity of FRA2 after CRP stimulation. Moreover, we used the target chromatin regions of Fra2 ChIP-seq data in mouse monocytes [36] and lifted them to human genome (see the “Methods” section). We found that FRA2 targeted genes were significantly upregulated in monocytes after CRP stimulation (Fig. 5f), further supporting that FRA2 is involved in the CRP-induced RA-associated chromatin dysregulation. For example, *OCSTAMP* and *IL-1B* as target genes of FRA2 and contributing to the RA pathogenesis [37, 38], their promoter regions became more accessible after CRP stimulation (Fig. 5g, h). Moreover, the mRNA expression of *OCSTAMP* and *IL-1B* were also significantly upregulated after CRP stimulation (Fig. 5i, j)*.* These findings suggested that CRP may induce RA-associated chromatin dysregulation through FRA2 in monocytes.

## Discussion

In this study, we surveyed the major immune cell types in the peripheral blood of RA patients. Although the main disease symptoms of RA appear at the joints, the recruitment of immune cells from the circulation is known to be essential for the progression of RA. As an autoimmune disease, the immunopathology of RA is characterized by autoantibodies and infiltration of T cells, B cells, and monocytes at the joints. Monocytes in RA spontaneously produce cytokines such as TNF-α, IL-1β, and IL-6, which inflame the articular joints and thereby amplify the function of osteoclasts. Moreover, monocytes themselves are able to differentiate into osteoclasts and contribute to cartilage damage [39]. Consistently, our study revealed that chromatin changes in monocytes dominated the RA-associated chromatin dysregulation in peripheral blood, which exhibited proinflammation and osteoclast differentiation features. Recently, droplet-based single-cell ATAC-seq (scATAC-seq) [40, 41] has become an powerful tool for genomic studies, enabling the identification of cell type-specific regulatory elements and disease-associated regulatory networks at single-cell resolution [40, 42]. The application of single-cell techniques can help researchers to reveal the details of regulome heterogeneities and discover regulatory elements in cell subtypes, such as monocyte subtypes, that are important in regulating the pathogenesis of rheumatoid arthritis; this provides new insights into the aetiology and potential therapies of the disease.

We found that the serum CRP level of RA patients is positively correlated with the extent of chromatin dysregulation in peripheral monocytes. Direct stimulation of OA patient-derived PBMCs with CRP also led to RA-like chromatin states. CRP is an important diagnostic biomarker of RA, which functions to promote the inflammation and bone destruction in RA [11, 27]. Since the production of CRP is induced by inflammatory cytokines such as TNF-α, IL-1β, and IL-6 [6, 7], we guess that these cytokines may also be involved in shaping the chromatin dysregulation we observed in the peripheral monocytes of RA patients. Consistent with this, it has been reported that inflammatory cytokines are capable of promoting epigenetic changes in RA patient monocytes [43]. Figure 2e shows that the strongest “hit” in the C3 area is “viral infectious disease”, but viral infection does not cause the serum CRP level to rise, which also shows that there are other factors in RA patients that affect monocyte chromatin dysregulation.

Our study illustrated FRA2 is involved in the CRP-induced RA-associated chromatin dysregulation. FRA2 belongs to the AP-1 TF family, which is composed of subunits of DNA-binding proteins from four families that all have similar DNA-binding motifs, including the Jun family, Fos family, ATF/CREB family, and Maf family [44]. Typically, AP-1 TFs form heterodimers or homodimers to function in a variety of cellular processes such as transformation, apoptosis, proliferation, and differentiation. Our study combined chromatin and RNA data and suggested that FRA2 may involve in regulation of the CRP-stimulated chromatin dysregulation. However, determining whether FRA2 forms homodimers or heterodimers with other TFs to regulate the downstream effects of CRP-induced, RA-associated chromatin dysregulation in peripheral monocytes will require additional experiments.

Given the known essential function of monocytes in RA pathogenesis, it makes sense that drugs targeting monocyte-associated proinflammatory signal pathways generally confer good clinical efficacies for RA treatment. For example, monoclonal antibodies against TNF (e.g. etanercept, infliximab, golimumab, adalimumab) and against IL-6R (e.g. tocilizumab, sarilumab) can relieve the disease in 20–22% of methotrexate insufficient responders. Drugs that target biomolecules downstream of the JAK-STAT pathway (e.g. tofacitinib, baricitinib) also confer similar efficacy [12]. Besides inflammation, OC-differentiation of monocytes is another reason for bone erosion in RA. However, it is reported that RA patients with high levels of serum CRP exhibit limited efficiency for immunosuppressive drugs such as Leflunomide and still develop progressive bone erosion [45]. In the present study, we found that CRP promotes RA pathogenesis via inducing RA-associated chromatin dysregulation that involved in both proinflammatory and OC-differentiation processes in peripheral monocytes, suggesting that targeting FRA2 could help protect against the aberrant activation of downstream genetic networks that contribute to RA pathogenesis following CRP-mediated chromatin dysregulation.

## Conclusions

In summary, we profiled the main immune cell types in the peripheral blood of RA and OA patients and healthy donors using ATAC-seq and identified the RA-associated chromatin dysregulation in monocytes as an RA signature. We found that the RA-associated chromatin dysregulation is related to the serum CRP level of RA patients and can be induced by CRP stimulation in vitro. Furthermore, we found FRA2 as a key transcription factor, which is responsible for the CRP induced RA-associated chromatin dysregulation. This RA-associated dysregulation pathway and related regulators could be potential therapeutic targets for RA.

## Methods

### Study design

The objective of this study was to use ATAC-seq to identify the RA-associated chromatin dysregulation signature in immune cells from RA patients, OA patients, and healthy donors. We collected fresh peripheral blood from OA and RA patients with heparin sodium anticoagulation tubes and obtained peripheral blood mononuclear cells (PBMCs) using Ficoll-Paque gradient centrifugation (Solarbio Science & Technology Co., Ltd., Cat No. P8900). Among them, 26 RA samples and 23 OA samples were used for ATAC-seq library construction; 8 OA samples were used for in vitro CRP stimulation experiments (5 for ATAC-seq, 3 for RNA-seq). 6 OA samples were used for qPCR in vitro CRP stimulation experiments; 8 RA samples and 5 OA samples were examined with flow cytometry (SONY 800S). Details on the sample collection and processing are described in the “Methods” section. Additionally, we downloaded data of 14 healthy donors (HDs) from the GEO database (GSE118189 [17], GSE74912 [18]) as healthy controls.

### Patients recruit

We recruited RA patients (all patients were treatment naive) and OA patients matched for age and sex from December 2017 to May 2020; all donors received oral and written information about the possibility that their blood would be used for research purposes and gave written consent. All of the RA patients met the 1987 American College of Rheumatology (ACR) classification criteria [46]. The sample collection procedures passed the ethical review for human biomedical research of the University of Science and Technology of China (USTCEC201700012). Samples were obtained from the First Affiliated Hospital of the University of Science and Technology of China. The clinical data collected for the patients are summarized in Additional file 2: Table S1.

### Flow cytometry and cell sorting

We collected fresh peripheral blood from OA and RA patients with heparin sodium anticoagulation tubes and obtained peripheral blood mononuclear cells (PBMCs) using Ficoll-Paque gradient centrifugation. PBMCs were stained with fluorochrome-labelled anti-human monoclonal antibodies (Biolegend Inc., San Diego, CA) to CD45 (clone HI30, Cat No. 304014 / 304007 / 304005), CD14 (clone HCD14, Cat No. 325620), CD19 (clone SJ25C1, Cat No. 363006), CD3 (clone OKT3, Cat No. 317343), CD4 (clone RPA-T4, Cat No. 300538), CD8α (clone RPA-T8, Cat No. 301008), and CD16 (clone 3G8, Cat No. 302008) for 15 min at room temperature, followed by DAPI (Cat No. 422801) staining for 10 min. Using a flow cytometer, antibody-stained patient lymphocytes were sorted into monocytes (DAPI-CD45+CD3+CD19-CD14+), B cells (DAPI-CD45+CD3-CD19+), CD4+ T cells (DAPI-CD45+CD3+CD19-CD4+CD8-), and CD8+ T cells (DAPI-CD45+CD3+CD19-CD4+CD8+). At least 50,000 cells were enriched. Post-sort purities of each cell type were ensured for > 95% with flow cytometry. For monocyte subpopulation analysis, monocytes were classified as classical (DAPI-CD45+CD3+CD19-CD14++CD16-), intermediate (DAPI-CD45+CD3+CD19-CD14++CD16+), and non-classical (DAPI-CD45+CD3+CD19- CD14+CD16+).

### ATAC-seq library

ATAC-seq was performed as previously described [16, 47]. Libraries were sequenced with the Illumina HiSeq X Ten platform; at least 10 million (18 million on average) paired-end reads were generated per sample. The quality control (QC) table of all analysed data is listed in Additional file 3: Table S2.

### RNA-seq library

Monocytes (DAPI-CD45+CD3+CD19-CD14+) were sorted by flow cytometry using the same sorting strategy described above. For monocytes, up to 1000 cells were collected directly in the 0.2 ml PCR tube (KIRGEN Bioscience Inc., Cat No. KG2511) and the RNA-seq library was constructed using Smart-seq2 method [48] and sequenced on the Illumina HiSeq X Ten platform; at least 20 million paired reads were generated per sample.

### In vitro stimulation of monocytes with CRP

PBMCs from OA patients were isolated using Ficoll-Paque gradient centrifugation and then stimulated with human recombinant CRP (0 μg/mL, 10 μg/mL) (R&D Systems Europe Ltd. Cat No.1707-CR-200/CF). Cells were cultured for 12 h in Roswell Park Memorial Institute (RPMI) 1640 medium supplemented with 10% of heat-inactivated human serum. After 12 h, control and stimulation cells were harvested and monocytes were isolated by flow cytometry using the same sorting strategy we used for the RA peripheral blood samples. Finally, ATAC-seq and RNA-seq library construction and sequencing was performed using the same strategies described above.

### Analysis of flow cytometry data

Flow cytometer data were analysed using the FlowJo V.X.0.7 software (Tree Star). Statistical analyses and approximations were done with GraphPad Prism 7 software (GraphPad Software Inc., USA).

### Real-time quantitative polymerase chain reaction

The selected candidate genes were validated by qPCR. Briefly, the cDNA was synthesized in accordance with the instructions indicated in a Maxima H Minus Reverse Transcriptase (ThermoFisher Scientific, USA, Cat No. EP0751). Two-step PCR was performed by using SYBR Green PCR Master Mix (Applied Biosystems, USA, Cat No.4344463) in accordance with the instructions of the manufacturer. The reaction was run on an LightCycler96 fluorescent sequence detection system (Roche). Gene expression was quantified relative to the expression of the housekeeping gene *GAPDH* and normalized to control by standard 2^-∆∆CT^ calculation. Primer sequences of gene *GAPDH* used are 5′-GGAGCGAGATCCCTCCAAAAT-3′ and 5′-GGCTGTTGTCATACTTCTCATGG-3′; primer sequences of gene *FOSL2* used are 5′-CAGAAATTCCGGGTAGATATGCC-3′ and 5′-GGTATGGGTTGGACATGGAGG-3′; primer sequences of gene *IL-1B* used are: 5′-AGCTACGAATCTCCGACCAC-3′ and 5′-CGTTATCCCATGTGTCGAAGAA-3′; primer sequences of gene *OCSTAMP* used are 5′-CACCCTGGGTATGGAGCAG-3′ and 5′-CTGGTGAGTGGTATTGAGGAGA-3′.

### Primary data processing and peak calling of ATAC-seq

ATAC-seq raw data was processed using a published ATAC-seq pipeline called ATAC-pipe [19]. After accounting for adapters, we used the “--MappingQC” function in the ATAC pipeline (option -c 50) to map high-quality reads to the hg19 genome using Bowtie2 [49]. PCR duplicates were removed and mapped reads were then shifted + 4/− 5 bp depending on the strand of the read, so that the first base of each mapped read represented the Tn5 cleavage position. For samples with biological replicates, we merged the fastq files as a sample for subsequent data processing. All mapped reads were then extended to 50 bp, centred by the cleavage position. Reads mapped to repeated regions and chromosome M were removed. The peaks calling steps used were based on the previously published ATAC-pipe “--PeakCalling” with options --p1 3 --q1 5 --f1 1 -u --pipeup 30. Samples from the same cell type classified within the same clinical condition (i.e. HD, OA, or RA) were grouped for peak calling, and peaks for all categories were then merged together to generate a unique peak list. We used quantile normalization to normalize the raw read counts after removing chromosome Y and to generate log_2_ peak intensity for downstream analysis.

### Primary data processing of RNA-seq

After removing low quality and adapter sequences, the remaining clean reads were aligned to the hg19 genome using STAR [50, 51]. We used bedtools “genomeCoverageBed” function to get a bedGraph file with histogram of coverage values [52].

### Differential analysis

For ATAC-seq, each cell type was compared with all other cell types, and cell type-specific peaks were filtered with |log_2_ fold change| > 4, *p* < 0.001, FDR < 0.01 and the average of log_2_(peak counts) > 3 across all samples. For each cell type, a pairwise comparison among HD, OA, and RA samples was performed, and disease-specific peaks were filtered with |log_2_ fold change| > 1, p < 0.001, and FDR < 0.1. An unpaired Student’s *t*-test and Benjamini-Hochberg multiple test were used to calculate the *p* and FDR values between any pair of samples. Unsupervised clustering of differential peaks is performed using K-means algorithm. For ATAC-seq stimulated by CRP in vitro, we aligned the fastq data to the hg19 genome according to the ATAC-pipe described above. We then used the ATAC-seq peaks of monocytes from the RA, OA, and HD samples as input and calculated the peak intensity in the CRP-stimulated samples. Differential peaks were filtered by fold change > 1.5, *p* < 0.01. *P* is calculated using paired *t*-test. For RNA-seq, we used raw read counts mapped to each gene as input and used DEseq2 [53] to obtain a standardized expression matrix. The differential genes were filtered as fold change > 1.5, *p* < 0.05, and the sum of counts across all samples is greater than 5. *P* is calculated using independent sample unpaired *t*-test.

### Genome segmentation analysis

The genome location classification for monocytes was performed using ChromHMM according to the Epigenomic Roadmap Consortium [54]. The chromatin states were classified with ChromHMM 25-state classification and then combined into 10 states, namely, “activate enhancer”, “primed enhancer”, “poised enhancer”, “weak enhancer”, “Tx regulator”, “promoter”, “transcription”, “quiescent/low”, “heterochromatin”, and “other”. The chromatin states of peaks were annotated by intersecting the ChromHMM-defined states using bedtools.

### Functional enrichment analysis by Metascape

To perform the biology function enrichment analysis of differential expression gene, we used the Metascape [30] with the default parameters. The terms with –log_10_ *p* > 4.5 were selected.

### Functional enrichment and associated genes analysis by GREAT

The significant differential peaks were uploaded to GREAT (version 3.0.0, Human GRCh37) for functional annotations [20]. Enrichment analyses of Biological Processes, Disease Ontologies, and MSigDB pathways of the peaks were performed using GREAT with the default options of “Basic Plus Extended Model”. Genes output by GREAT were taken as peak-related genes.

### Peak cluster score calculation

Cluster scores of C1-C3 were calculated by dividing the sum of the peak intensities of each cluster by the sum of the total peak count, multiplied by 100.The C3 score was defined as RA-associated ATAC-seq score (RAAS).

### Calculation of the relationship between RAAS and patient clinical data

The “OLS” function in the statsmodels Python package was used to perform linear regression as an approach to measure the correlation of RAAS with the clinical data of RA patients including DAS28-CRP, DAS28-ESR, CRP, ESR, TCJ, SCJ, RF, and Anti-CCP. *R*-squared is a goodness-of-fit measure for linear regression models, and *p* characterizes the probability that two variables are significantly linearly related.

### GSEA enrichment analysis

We first arranged the gene matrix of log_2_ fold change (CRP^stim^/control) in descending order and then use the R package GSEABase to calculate the enrichment score and *p* of the gene set downloaded from MSigDB [28] in the gene matrix. The values of *p* less than 0.05 are considered to be significantly enriched. “Proinflammatory gene set” and “regulation of OC Differentiation gene set” are available from MSigDB with ID GO:0050729 (https://www.gsea-msigdb.org/gsea/msigdb/cards/GO_POSITIVE_REGULATION_OF_INFLAMMATORY_RESPONSE) and GO:0045670 (http://www.gsea-msigdb.org/gsea/msigdb/geneset_page.jsp?geneSetName=GO_REGULATION_OF_OSTEOCLAST_DIFFERENTIATION).

### TF motif analysis

The input motif set we used was obtained from jaspar (http://jaspar.genereg.net/) for vertebrates. We searched for enriched motifs in differentially accessible regions using the “findMotifsGenome.pl” script in HOMER [35]. We generated a peak versus motif matrix, where each row is a peak and each column is a motif. We applied these two matrixes to Genomica [55], as input for the ModuleMap algorithm. Then, we obtained a motif-by-sample matrix, where each row is a motif, each column is a sample, and the values in this matrix represent the significance of enrichment by the –log (*p* value).

### TF foot-printing analysis

TF foot-printing analysis was processed using ATAC-pipe. To analyse the footprint of each TF, ATAC-pipe function “--Footprint” scans TF-binding sites by invoking motif matrix with HOMER, filters out sites with CRP stimulation significant changes peak list to obtain the TF-binding sites, and counts for the per base Tn5 cleavage events around the centres of TF-binding sites (− 100 to 100 bp).

### ChIP-seq analysis

ChIP-seq data on Fra2 of mouse monocytes were obtained from previously published articles [36]. We used UCSC [56] lifting tool liftover (http://genome.ucsc.edu/cgi-bin/hgLiftOver) to convert genome position from mm10 genome assembly to hg19 genome assembly [56]. Then, we used GREAT to find the target genes of FRA2 (explained in detail in the “Annotating ATAC-seq peak regions” method).

### Statistics

To analyse the significant change in cluster peak scores stimulated by CRP, we used paired Student’s *t* test. Fisher’s exact test is used to calculate *p* to test the significance of associations between gene sets. The remaining data in this study were assessed using unpaired Student’s *t* tests. The levels of significance were indicated as follows: *p < 0.05, **p < 0.01, ***p < 0.001, and ****p < 0.0001.

## Supplementary Information

**Additional file 1: Figure S1.** Immune cell sorting strategy and quality control of ATAC-seq profiles. **a** Gating strategy for flow cytometry. **b** Purity of cells after sorting by flow cytometry. **c** Repeatability between two replicates for ATAC-seq data. **d** Within 2 kb of the promoter, the reads in the ATAC-seq data were concentrated in the TSS region. **e** Analysis of ATAC-seq data to display representative cell markers of immune cells. OA, osteoarthritis; RA, rheumatoid arthritis. **Figure S2.** The chromatin accessibilities profiles for immune cells from OA patients, RA patients, and healthy donors. **a** Flow cytometry analysis of the proportion of monocyte subpopulations in patients with OA (*n* = 5) and RA (*n* = 8). *P* values were assessed using an unpaired Student’s t-test, ns: *p* > 0.05. Error bars represent standard error of the mean (SEM). **b** Heatmap of chromatin dysregulation peaks obtained by comparing the ATAC-seq profiles of B cells and T cells from HDs, OA patients, and RA patients (|log2FD| > 1, *p* < 0.001 and FDR < 0.1). Each column is a sample; each row is a dysregulated chromatin region. The elements were organized based on unsupervised clustering. **c** Representation of selected top disease ontology categories obtained from the analysis of Cluster 1–2 regions using GREAT. Samples from the same group are marked with the same colour. HD, healthy donors; OA, osteoarthritis; RA, rheumatoid arthritis; C1, cluster 1; C2, cluster 2; C3, cluster 3. **Figure S3.** The functional genomic characteristics of Cluster 3. **a** Normalized ATAC-seq profiles at the *IL-1B* and *JAK1* loci in HD, OA and RA. Shaded regions indicate peaks that are more accessible in RA patients. **b** Distribution of genomic features of Cluster 3 peaks. Different genomic features are annotated with different colours. HD, healthy donor; OA, osteoarthritis; RA, rheumatoid arthritis. **Figure S4.** Principal component analysis based on the Cluster 1 (a) and Cluster 2 (b) regions for HDs, OA patients, and RA patients. Each dot is a sample, the samples in the figure are coloured by disease states. HD, healthy donors; OA, osteoarthritis; RA, rheumatoid arthritis. **Figure S5.** The relationship between peak clusters and the clinical status of patients. **a**, **b** Linear regression analysis was used to correlate RAAS with DAS28-ESR (**a**), TCJ, SCJ, RF, ESR, and Anti-CCP(**b**). The shading areas represent the 95% confidence intervals. The solid line was fit from linear regression, and the *p* value and the square of the coefficient of correlation (R^2^) were calculated using the ‘OLS’ function in the statsmodels package in Python. P value < 0.05 were considered as significant. DAS28_ESR: disease activity score DAS28 based on erythrocyte sedimentation rate; SJC, joint swelling count; TJC, tender joint count; ESR, erythrocyte sedimentation rate; Anti-CCP, anti-cyclic citrullinated peptide; RF, rheumatoid factor; RAAS, RA-associated ATAC-seq score. **Figure S6.** Quality control of RNA-seq and ATAC-seq of CRP stimulated monocytes in vitro. **a** Within 2kb of the promoter, the reads in the ATAC-seq data were concentrated in the TSS region. **b** Repeatability between two replicates for ATAC-seq data. **c** Box plot showing the distribution of normalized counts for RNA-seq data. Each bar represents a sample. **d** Principal component analysis of CRP^stim^ and control group based on expression of all genes. Each point is a sample, and the samples in the figure are coloured by groups. CRP^stim^, CRP stimulation. **Figure S7.** CRP stimulation promotes OC-differentiation and proinflammation in OA-derived monocytes. **a** Heatmap showing changes in gene expression in monocytes stimulated with CRP (10 μg/mL) for 12 hours (paired t-test *p* < 0.05 and fold change > 2). RNA-seq was performed for three independent biological replicates (monocytes derived from three different patients with OA). (right) Metascape was used to annotate genes; enriched GO terms after CRP stimulation. **b**, **c** Heatmaps show the expression levels of genes in the proinflammation (b) and regulation of OC-differentiation (c) gene sets. **d** Heatmap showing the expression levels of the genes of cluster 3 regions annotated by GREAT (identified in Fig. 2c). CRP^stim^, CRP stimulation; OC, osteoclast; C3, cluster 3. **Figure S8.** The functional genomic characteristics of differential regions after CRP stimulation. **a** Enriched GO terms of genes related to the peaks upregulated after CRP stimulation by GREAT. **b** Normalized ATAC-seq profiles at the *CSF1R* and *SCIN* loci in the control and CRP^stim^ groups. Shaded regions indicate peaks that are more accessible in CRP^stim^. **c** Enriched GO terms among genes related to the peaks that became less accessible after CRP stimulation by GREAT. **d** Distribution of genomic features in the upregulated (left) and downregulated (right) regions after CRP stimulation. Different genomic features are annotated with different colours. **Figure S9.** Transcription factor motifs enrich in cluster 3. **a** Enrichment of transcription factor motifs in C3 for all samples. Each row is a motif, and each column is a sample. Values in the matrix indicate the significance of enrichment estimated by Genomica in terms of –Log_10_ p. The top ranked motifs are shown. The colour bar indicates the category of each sample: HD, healthy donor; OA, osteoarthritis; RA, rheumatoid arthritis. **b** Transcription factor motifs enriched in the Cluster 3 peaks using HOMER and ranked by p value.

**Additional file 2: Table S1.** List of information for patients with RA and OA.

**Additional file 3: Table S2.** QC table for all analysed ATAC-seq samples.

**Additional file 4: Table S3.** The list of differentially accessible regions in monocytes among RA, OA, and healthy donors.

**Additional file 5: Table S4.** The list of C3 related genes.

**Additional file 6: Table S5.** The list of significantly changed chromatin accessible regions and peak related genes in OA-patient-derived monocytes after CRP stimulation.

**Additional file 7: Table S6.** The list of differential expressed genes in OA-patient-derived monocytes after CRP stimulation.

## Data Availability

The raw ATAC-seq data of RA and OA samples used in this study have been deposited in the Genome Sequence Archive (GSA, https://bigd.big.ac.cn/gsa) [57] in National Genomics Data Center [58], under accession number CRA002749, that are publicly accessible at https://bigd.big.ac.cn/gsa/browse/CRA002749. Other published ATAC-seq data sets used in this study are available from the Gene Expression Omnibus (GEO, https://www.ncbi.nlm.nih.gov/geo/) database (accession number GSE118189 [17] for Monocytes: https://identifiers.org/geo:GSE118189; GSE74912 [18] for B cells, CD4+ T cells, and CD8+ T cells: https://identifiers.org/geo:GSE74912). ChIP-seq data of Fra2 are available from GEO database (accession number GSE111854 [36]: https://www.ncbi.nlm.nih.gov/geo/query/acc.cgi?acc=GSE111854). The datasets supporting the conclusions of this article are included within the article and its additional files (see Additional file 2-7). All relevant codes are available through the GitHub repository (https://github.com/QuKunLab/RA-OA).

## Abbreviations

AP-1: Activator protein 1
C1: Cluster 1
C2: Cluster 2
C3: Cluster 3
CRP: C-reaction protein
CRP^stim^: CRP stimulation
DAS28-CRP: Disease activity score DAS28 based on C-reaction protein levels
DAS28-ESR: Disease activity score DAS28 based on the erythrocyte sedimentation rate
HD: Healthy donor
OA: Osteoarthritis
OC: Osteoclast
PBMC: Peripheral blood mononuclear cell
RA: Rheumatoid arthritis
RAAS: RA-associated ATAC-seq score

## References

[1] Myasoedova E, Crowson CS, Kremers HM, Therneau TM, Gabriel SE (2010). Is the incidence of rheumatoid arthritis rising?: results from Olmsted County, Minnesota, 1955-2007. Arthritis Rheum.

[2] McInnes IB, Schett G (2017). Pathogenetic insights from the treatment of rheumatoid arthritis. Lancet..

[3] McInnes IB, Schett G (2011). The pathogenesis of rheumatoid arthritis. N Engl J Med.

[4] Pap T, Korb-Pap A (2015). Cartilage damage in osteoarthritis and rheumatoid arthritis--two unequal siblings. Nat Rev Rheumatol.

[5] Redlich K, Smolen JS (2012). Inflammatory bone loss: pathogenesis and therapeutic intervention. Nat Rev Drug Discov.

[6] Hurlimann J, Thorbecke GJ, Hochwald GM (1966). The liver as the site of C-reactive protein formation. J Exp Med.

[7] Clyne B, Olshaker JS (1999). The C-reactive protein. J Emerg Med.

[8] Dessein PH, Joffe BI, Stanwix AE (2004). High sensitivity C-reactive protein as a disease activity marker in rheumatoid arthritis. J Rheumatol.

[9] Aman S, Paimela L, Leirisalo-Repo M, Risteli J, Kautiainen H, Helve T, Hakala M (2000). Prediction of disease progression in early rheumatoid arthritis by ICTP, RF and CRP. A comparative 3-year follow-up study. Rheumatology (Oxford).

[10] Lu J, Marnell LL, Marjon KD, Mold C, Du Clos TW, Sun PD (2008). Structural recognition and functional activation of FcgammaR by innate pentraxins. Nature..

[11] Kim KW, Kim BM, Moon HW, Lee SH, Kim HR (2015). Role of C-reactive protein in osteoclastogenesis in rheumatoid arthritis. Arthritis Res Ther.

[12] Aletaha D, Smolen JS (2018). Diagnosis and management of rheumatoid arthritis: a review. JAMA..

[13] Viatte S, Plant D, Raychaudhuri S (2013). Genetics and epigenetics of rheumatoid arthritis. Nat Rev Rheumatol.

[14] Nemtsova MV, Zaletaev DV, Bure IV, Mikhaylenko DS, Kuznetsova EB, Alekseeva EA, Beloukhova MI, Deviatkin AA, Lukashev AN, Zamyatnin AA (2019). Epigenetic changes in the pathogenesis of rheumatoid arthritis. Front Genet.

[15] de Andres MC, Perez-Pampin E, Calaza M, Santaclara FJ, Ortea I, Gomez-Reino JJ, Gonzalez A (2015). Assessment of global DNA methylation in peripheral blood cell subpopulations of early rheumatoid arthritis before and after methotrexate. Arthritis Res Ther..

[16] Buenrostro JD, Giresi PG, Zaba LC, Chang HY, Greenleaf WJ (2013). Transposition of native chromatin for fast and sensitive epigenomic profiling of open chromatin, DNA-binding proteins and nucleosome position. Nat Methods.

[17] Calderon D, Nguyen MLT, Mezger A, Kathiria A, Muller F, Nguyen V, et al. Landscape of stimulation-responsive chromatin across diverse human immune cells. Nat Genet 2019, 51(10):1494–1505. https://identifiers.org/geo:GSE118189. 10.1038/s41588-019-0505-9 PMC6858557 31570894

[18] Corces MR, Buenrostro JD, Wu B, Greenside PG, Chan SM, Koenig JL, et al. Lineage-specific and single-cell chromatin accessibility charts human hematopoiesis and leukemia evolution. Nat Genet 2016, 48(10):1193–1203. https://identifiers.org/geo:GSE74912. 10.1038/ng.3646 PMC5042844 27526324

[19] Zuo Z, Jin Y, Zhang W, Lu Y, Li B, Qu K (2019). ATAC-pipe: general analysis of genome-wide chromatin accessibility. Brief Bioinform.

[20] McLean CY, Bristor D, Hiller M, Clarke SL, Schaar BT, Lowe CB (2010). GREAT improves functional interpretation of cis-regulatory regions. Nat Biotechnol.

[21] Pascual V, Allantaz F, Arce E, Punaro M, Banchereau J (2005). Role of interleukin-1 (IL-1) in the pathogenesis of systemic onset juvenile idiopathic arthritis and clinical response to IL-1 blockade. J Exp Med.

[22] Rodig SJ, Meraz MA, White JM, Lampe PA, Riley JK, Arthur CD, King KL, Sheehan KCF, Yin L, Pennica D, Johnson EM, Schreiber RD (1998). Disruption of the Jak1 gene demonstrates obligatory and nonredundant roles of the Jaks in cytokine-induced biologic responses. Cell..

[23] Ernst J, Kellis M (2012). ChromHMM: automating chromatin-state discovery and characterization. Nat Methods.

[24] Matsui T, Kuga Y, Kaneko A, Nishino J, Eto Y, Chiba N, Yasuda M, Saisho K, Shimada K, Tohma S (2007). Disease Activity Score 28 (DAS28) using C-reactive protein underestimates disease activity and overestimates EULAR response criteria compared with DAS28 using erythrocyte sedimentation rate in a large observational cohort of rheumatoid arthritis patients in Japan. Ann Rheum Dis.

[25] Sharma A, Khan R, Gupta N, Sharma A, Zaheer MS, Abbas M, Khan SA (2018). Acute phase reactant, Pentraxin 3, as a novel marker for the diagnosis of rheumatoid arthritis. Clin Chim Acta.

[26] van Zanten JJCSV, Ring C, Carroll D, Kitas GD (2005). Increased C reactive protein in response to acute stress in patients with rheumatoid arthritis. Ann Rheum Dis.

[27] Hanriot D, Bello G, Ropars A, Seguin-Devaux C, Poitevin G, Grosjean S (2008). C-reactive protein induces pro- and anti-inflammatory effects, including activation of the liver X receptor alpha, on human monocytes. Thromb Haemost.

[28] Liberzon A, Subramanian A, Pinchback R, Thorvaldsdottir H, Tamayo P, Mesirov JP (2011). Molecular signatures database (MSigDB) 3.0. Bioinformatics..

[29] Feng X, Teitelbaum SL (2013). Osteoclasts: new insights. Bone Res.

[30] Zhou Y, Zhou B, Pache L, Chang M, Khodabakhshi AH, Tanaseichuk O, Benner C, Chanda SK (2019). Metascape provides a biologist-oriented resource for the analysis of systems-level datasets. Nat Commun.

[31] Subramanian A, Kuehn H, Gould J, Tamayo P, Mesirov JP (2007). GSEA-P: a desktop application for Gene Set Enrichment Analysis. Bioinformatics..

[32] Ivashkiv LB, Hu X (2003). The JAK/STAT pathway in rheumatoid arthritis: pathogenic or protective?. Arthritis Rheum.

[33] Kameda Y, Takahata M, Komatsu M, Mikuni S, Hatakeyama S, Shimizu T, Angata T, Kinjo M, Minami A, Iwasaki N (2013). Siglec-15 regulates osteoclast differentiation by modulating RANKL-induced phosphatidylinositol 3-kinase/Akt and Erk pathways in association with signaling adaptor DAP12. J Bone Miner Res.

[34] Naser A, Odeh AK, Sharp RC, Qasem A, Beg S, Naser SA. Polymorphisms in TNF Receptor Superfamily 1B (TNFRSF1B:rs3397) are Linked to Mycobacterium avium paratuberculosis Infection and Osteoporosis in Rheumatoid Arthritis. Microorganisms. 2019;7(12):646. 10.3390/microorganisms7120646 PMC6955732 31817071

[35] Heinz S, Benner C, Spann N, Bertolino E, Lin YC, Laslo P, Cheng JX, Murre C, Singh H, Glass CK (2010). Simple combinations of lineage-determining transcription factors prime cis-regulatory elements required for macrophage and B cell identities. Mol Cell.

[36] Fonseca GJ, Tao J, Westin EM, Duttke SH, Spann NJ, Strid T, et al. Diverse motif ensembles specify non-redundant DNA binding activities of AP-1 family members in macrophages. Gene Expression Omnibus. 2019; https://www.ncbi.nlm.nih.gov/geo/query/acc.cgi?acc=GSE111854. 10.1038/s41467-018-08236-0 PMC6345992 30679424

[37] Ishii T, Ruiz-Torruella M, Ikeda A, Shindo S, Movila A, Mawardi H, Albassam A, Kayal RA, al-Dharrab AA, Egashira K, Wisitrasameewong W, Yamamoto K, Mira AI, Sueishi K, Han X, Taubman MA, Miyamoto T, Kawai T (2018). OC-STAMP promotes osteoclast fusion for pathogenic bone resorption in periodontitis via up-regulation of permissive fusogen CD9. FASEB J.

[38] Schett G, Dayer JM, Manger B (2016). Interleukin-1 function and role in rheumatic disease. Nat Rev Rheumatol.

[39] Udalova IA, Mantovani A, Feldmann M (2016). Macrophage heterogeneity in the context of rheumatoid arthritis. Nat Rev Rheumatol.

[40] Lareau CA, Duarte FM, Chew JG, Kartha VK, Burkett ZD, Kohlway AS, Pokholok D, Aryee MJ, Steemers FJ, Lebofsky R, Buenrostro JD (2019). Droplet-based combinatorial indexing for massive-scale single-cell chromatin accessibility. Nat Biotechnol.

[41] Satpathy AT, Granja JM, Yost KE, Qi Y, Meschi F, McDermott GP (2019). Massively parallel single-cell chromatin landscapes of human immune cell development and intratumoral T cell exhaustion. Nat Biotechnol.

[42] Buenrostro JD, Wu B, Litzenburger UM, Ruff D, Gonzales ML, Snyder MP, Chang HY, Greenleaf WJ (2015). Single-cell chromatin accessibility reveals principles of regulatory variation. Nature..

[43] Rodriguez-Ubreva J, de la Calle-Fabregat C, Li T, Ciudad L, Ballestar ML, Catala-Moll F (2019). Inflammatory cytokines shape a changing DNA methylome in monocytes mirroring disease activity in rheumatoid arthritis. Ann Rheum Dis.

[44] Gazon H, Barbeau B, Mesnard JM, Peloponese JM (2017). Hijacking of the AP-1 signaling pathway during development of ATL. Front Microbiol.

[45] Liang C, Li J, Lu C, Xie D, Liu J, Zhong C, Wu X, Dai R, Zhang H, Guan D, Guo B, He B, Li F, He X, Zhang W, Zhang BT, Zhang G, Lu A (2019). HIF1alpha inhibition facilitates Leflunomide-AHR-CRP signaling to attenuate bone erosion in CRP-aberrant rheumatoid arthritis. Nat Commun.

[46] Arnett FC, Edworthy SM, Bloch DA, McShane DJ, Fries JF, Cooper NS (1988). The American Rheumatism Association 1987 revised criteria for the classification of rheumatoid arthritis. Arthritis Rheum.

[47] Buenrostro JD, Wu B, Chang HY, Greenleaf WJ (2015). ATAC-seq: a method for assaying chromatin accessibility genome-wide. Curr Protoc Mol Biol.

[48] Picelli S, Faridani OR, Bjorklund AK, Winberg G, Sagasser S, Sandberg R (2014). Full-length RNA-seq from single cells using Smart-seq2. Nat Protoc.

[49] Langmead B, Trapnell C, Pop M, Salzberg SL (2009). Ultrafast and memory-efficient alignment of short DNA sequences to the human genome. Genome Biol.

[50] Bolger AM, Lohse M, Usadel B (2014). Trimmomatic: a flexible trimmer for Illumina sequence data. Bioinformatics..

[51] Dobin A, Davis CA, Schlesinger F, Drenkow J, Zaleski C, Jha S, Batut P, Chaisson M, Gingeras TR (2013). STAR: ultrafast universal RNA-seq aligner. Bioinformatics..

[52] Quinlan AR, Hall IM (2010). BEDTools: a flexible suite of utilities for comparing genomic features. Bioinformatics..

[53] Love MI, Huber W, Anders S (2014). Moderated estimation of fold change and dispersion for RNA-seq data with DESeq2. Genome Biol.

[54] Roadmap Epigenomics C, Kundaje A, Meuleman W, Ernst J, Bilenky M, Yen A (2015). Integrative analysis of 111 reference human epigenomes. Nature..

[55] Segal E, Friedman N, Koller D, Regev A (2004). A module map showing conditional activity of expression modules in cancer. Nat Genet.

[56] Kent WJ, Sugnet CW, Furey TS, Roskin KM, Pringle TH, Zahler AM (2002). The human genome browser at UCSC. Genome Res.

[57] Wang Y, Song F, Zhu J, Zhang S, Yang Y, Chen T, Tang B, Dong L, Ding N, Zhang Q, Bai Z, Dong X, Chen H, Sun M, Zhai S, Sun Y, Yu L, Lan L, Xiao J, Fang X, Lei H, Zhang Z, Zhao W (2017). GSA: genome sequence archive<sup/>. Genomics Proteomics Bioinformatics.

[58] Yong X, Yi B, Zhang Z, Wen Z, Jing X, Shun H, et al. China National Center for Bioinformation in 2021. Nucleic Acids Res. 2021;49(D1):D18–28. 10.1093/nar/gkaa1022 PMC7779035 33175170

