# Supplementary material for: Chromatin accessibility landscapes of immune cells in rheumatoid arthritis nominate monocytes in disease pathogenesis
Source: BMC Biol. 2021 Apr 16;19:79. doi: 10.1186/s12915-021-01011-6 (PMC8050920; doi:10.1186/s12915-021-01011-6)
Supplement: Supplementary file 1 — Additional file 1: Figure S1. Immune cell sorting strategy and quality control of ATAC-seq profiles. a Gating strategy for flow cytometry. b Purity of cells after sorting by flow cytometry. c Repeatability between two replicates for ATAC-seq data. d Within 2 kb of the promoter, the reads in the ATAC-seq data were concentrated in the TSS region. e Analysis of ATAC-seq data to display representative cell markers of immune cells. OA, osteoarthritis; RA, rheumatoid arthritis. Figure S2. The chromatin accessibilities profiles for immune cells from OA patients, RA patients, and healthy donors. a Flow cytometry analysis of the proportion of monocyte subpopulations in patients with OA (n = 5) and RA (n = 8). P values were assessed using an unpaired Student’s t-test, ns: p > 0.05. Error bars represent standard error of the mean (SEM). b Heatmap of chromatin dysregulation peaks obtained by comparing the ATAC-seq profiles of B cells and T cells from HDs, OA patients, and RA patients (|log2FD| > 1, p < 0.001 and FDR < 0.1). Each column is a sample; each row is a dysregulated chromatin region. The elements were organized based on unsupervised clustering. c Representation of selected top disease ontology categories obtained from the analysis of Cluster 1–2 regions using GREAT. Samples from the same group are marked with the same colour. HD, healthy donors; OA, osteoarthritis; RA, rheumatoid arthritis; C1, cluster 1; C2, cluster 2; C3, cluster 3. Figure S3. The functional genomic characteristics of Cluster 3. a Normalized ATAC-seq profiles at the IL-1B and JAK1 loci in HD, OA and RA. Shaded regions indicate peaks that are more accessible in RA patients. b Distribution of genomic features of Cluster 3 peaks. Different genomic features are annotated with different colours. HD, healthy donor; OA, osteoarthritis; RA, rheumatoid arthritis. Figure S4. Principal component analysis based on the Cluster 1 (a) and Cluster 2 (b) regions for HDs, OA patients, and RA patients. Each dot is a sa [file 12915_2021_1011_MOESM1_ESM.pdf]

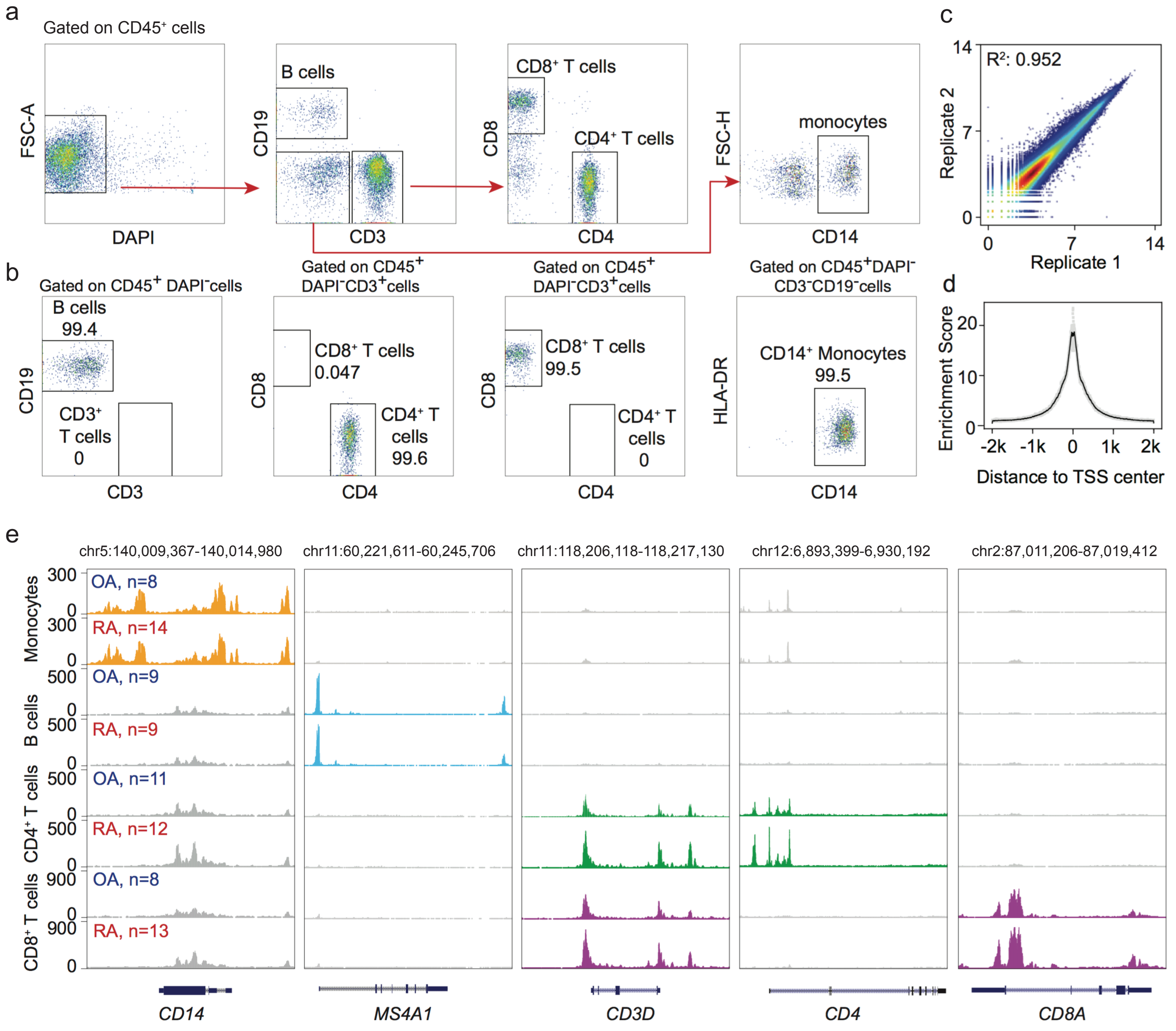


**Fig. S1 Immune cell sorting strategy and quality control of ATAC-seq profiles. a** Gating strategy for flow cytometry. **b** Purity of cells after sorting by flow cytometry. **c** Repeatability between two replicates for ATAC-seq data. **d** Within 2kb of the promoter, the reads in the ATAC-seq data were concentrated in the TSS region. **e** Analysis of ATAC-seq data to display representative cell markers of immune cells. OA, osteoarthritis; RA, rheumatoid arthritis.

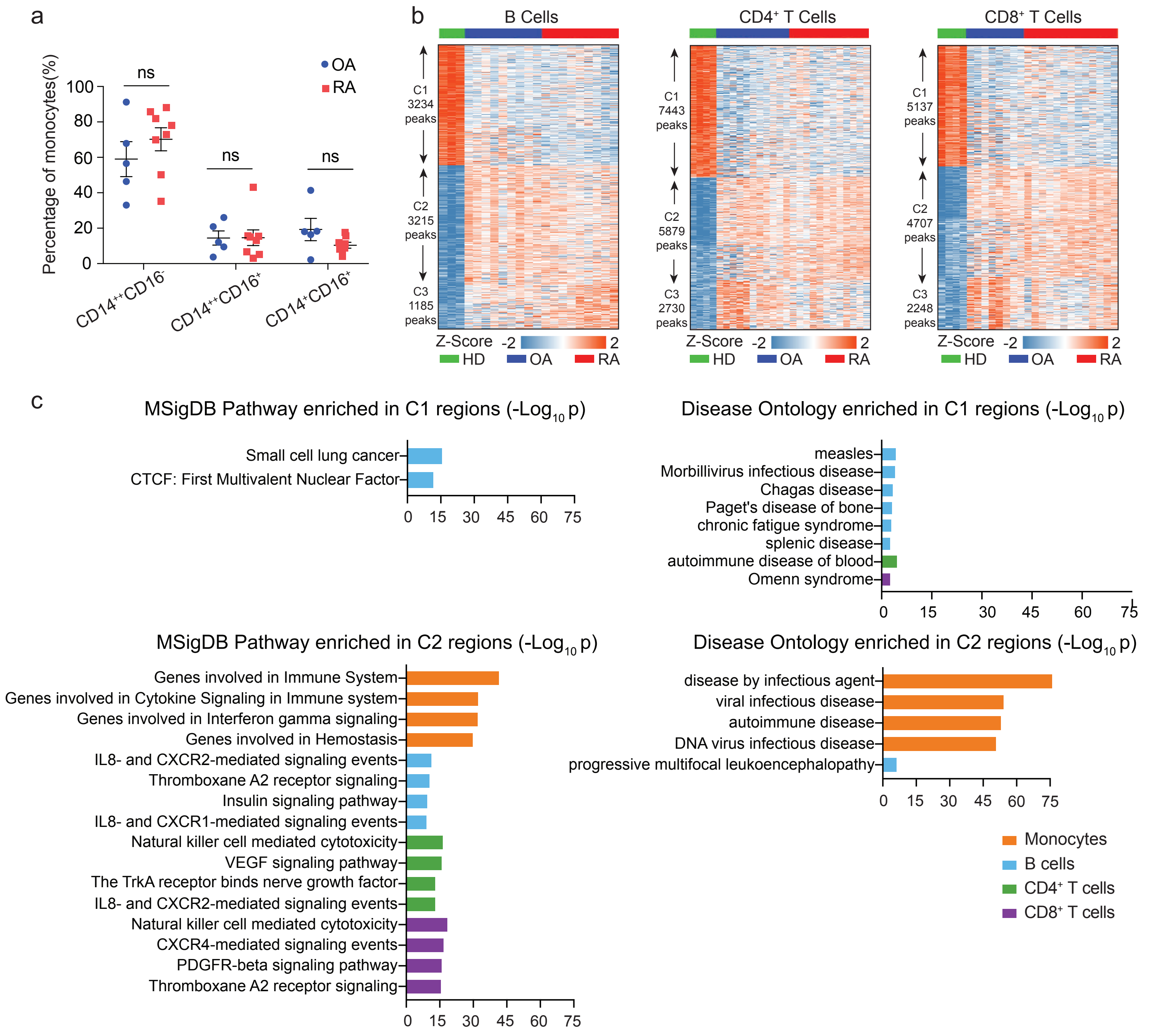


**Fig. S2 The chromatin accessibilities profiles for immune cells from OA patients, RA patients, and healthy donors. a** Flow cytometry analysis of the proportion of monocyte subpopulations in patients with OA (n=5) and RA (n=8). P values were assessed using an unpaired Student's t-test, ns: $p > 0.05$. Error bars represent standard error of the mean(SEM). **b** Heatmap of chromatin dysregulation peaks obtained by comparing the ATAC-seq profiles of B cells and T cells from HDs, OA patients, and RA patients (|log2FD| > 1, $p < 0.001$ and FDR < 0.1). Each column is a sample; each row is a dysregulated chromatin region. The elements were organized based on unsupervised clustering. **c** Representation of selected top disease ontology categories obtained from the analysis of Cluster 1-2 regions using GREAT. Samples from the same group are marked with the same colour. HD, healthy donors; OA, osteoarthritis; RA, rheumatoid arthritis; C1, cluster 1; C2, cluster 2; C3, cluster 3.

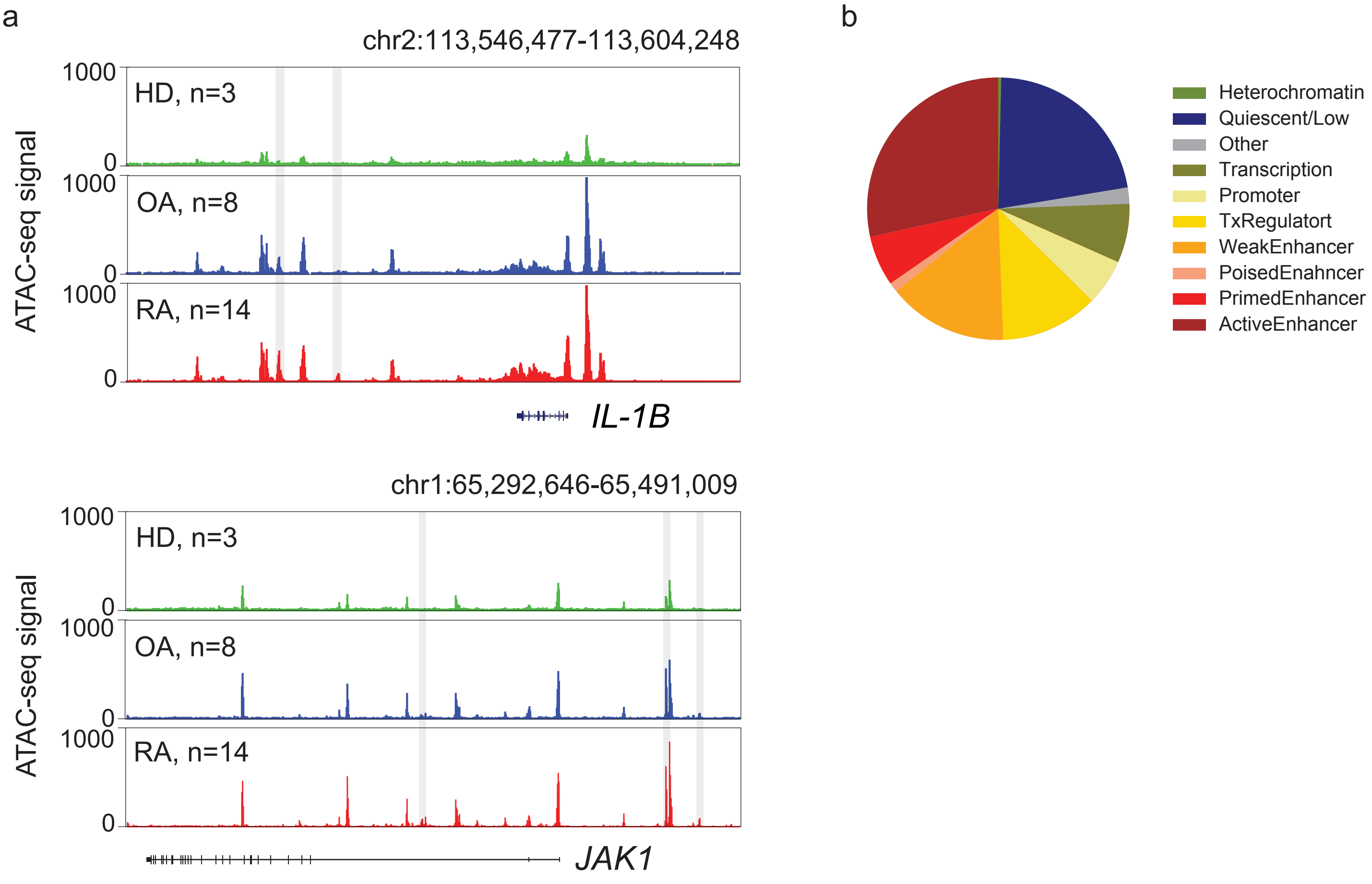


**Fig. S3 The functional genomic characteristics of Cluster 3. a** Normalized ATAC-seq profiles at the *IL-1B* and *JAK1* loci in HD, OA and RA. Shaded regions indicate peaks that are more accessible in RA patients. **b** Distribution of genomic features of Cluster 3 peaks. Different genomic features are annotated with different colours. HD, healthy donor; OA, osteoarthritis; RA, rheumatoid arthritis.

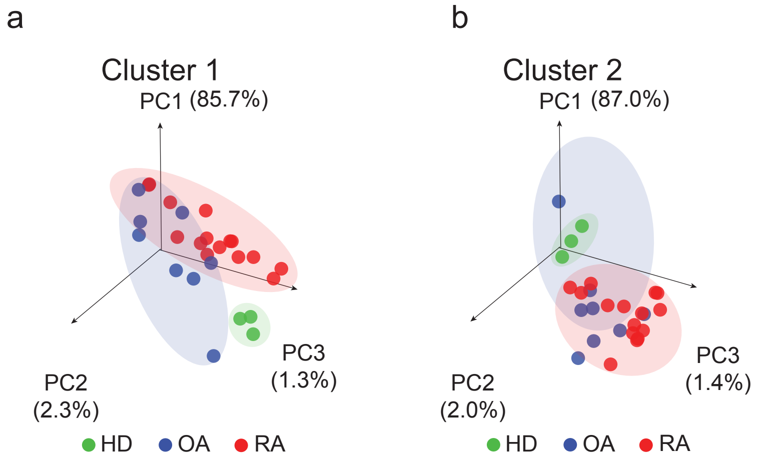


**Fig. S4 Principal component analysis based on the Cluster 1 (a) and Cluster 2 (b) regions for HDs, OA patients, and RA patients.** Each dot is a sample, the samples in the figure are coloured by disease states. HD, healthy donors; OA, osteoarthritis; RA, rheumatoid arthritis.

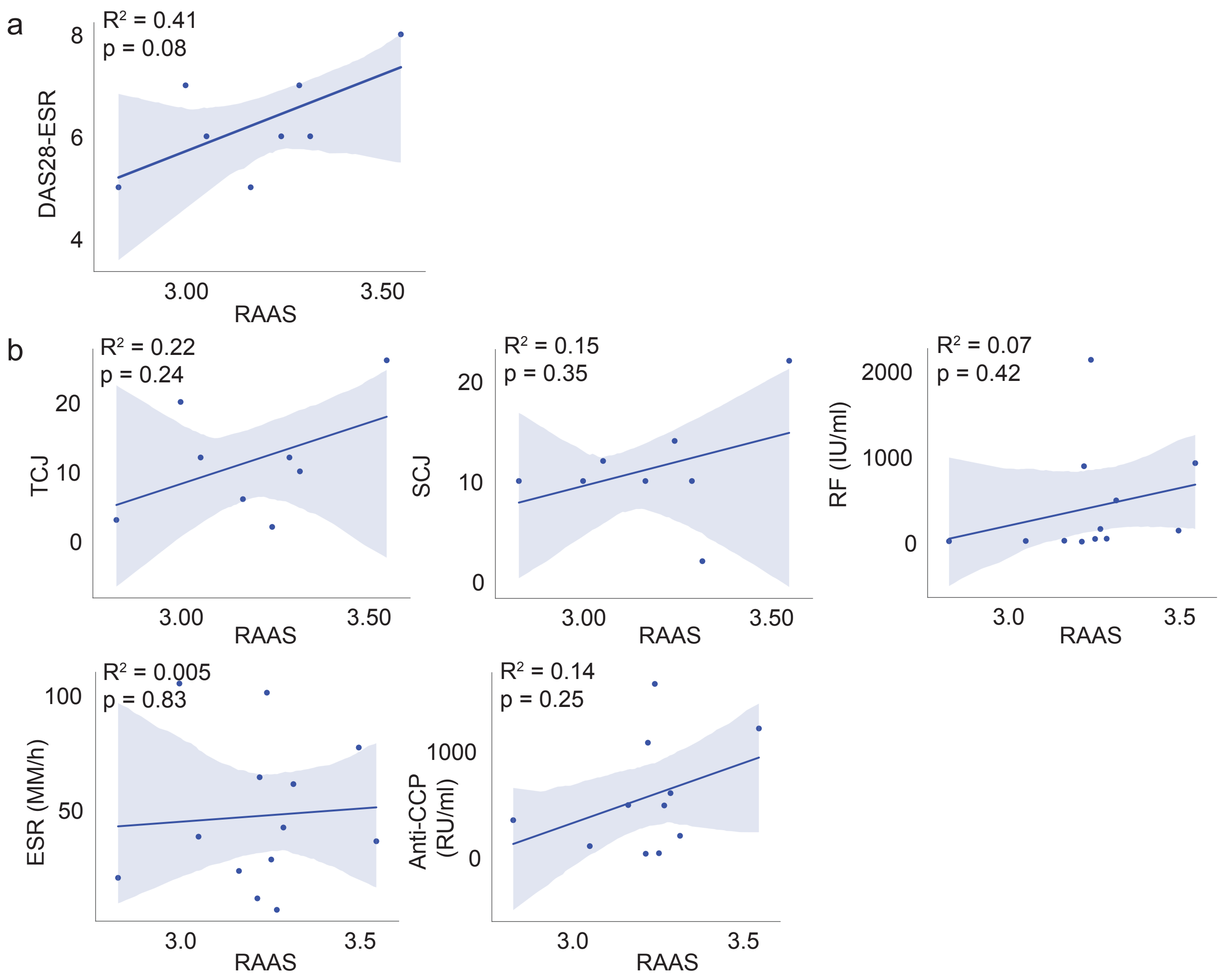


**Fig. S5 The relationship between peak clusters and the clinical status of patients. a**, **b** Linear regression analysis was used to correlate RAAS with DAS28-ESR (**a**), TCJ, SCJ, RF, ESR, and Anti-CCP(**b**). The shading areas represent the 95% confidence intervals. The solid line was fit from linear regression, and the p value and the square of the coefficient of correlation ($R^2$) were calculated using the 'OLS' function in the statsmodels package in Python. P value < 0.05 were considered as significant. DAS28_ESR: disease activity score DAS28 based on erythrocyte sedimentation rate; SJC, joint swelling count; TJC, tender joint count; ESR, erythrocyte sedimentation rate; Anti-CCP, anti-cyclic citrullinated peptide; RF, rheumatoid factor; RAAS, RA-associated ATAC-seq score.

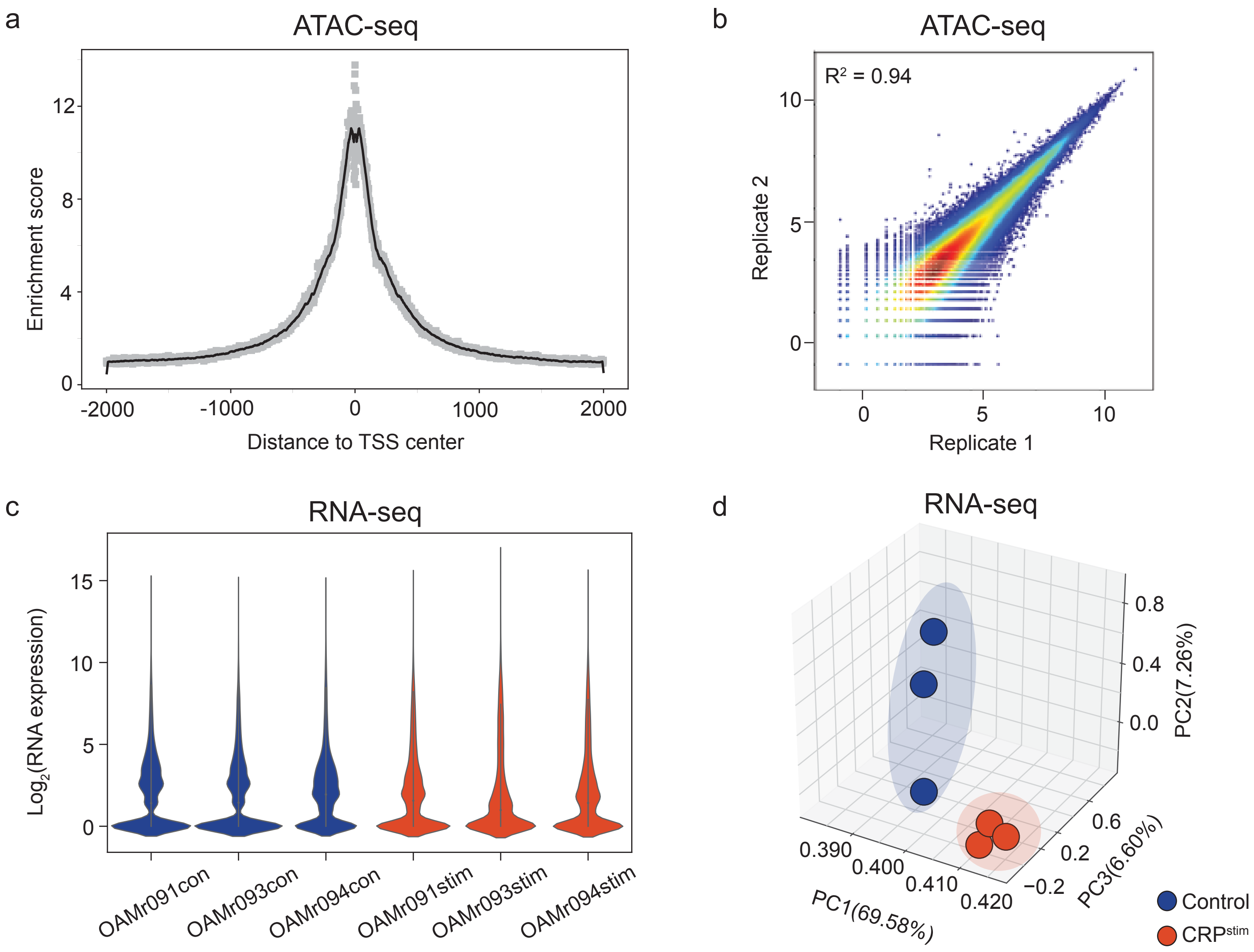


**Fig. S6 Quality control of RNA-seq and ATAC-seq of CRP stimulated monocytes *in vitro*. a** Within 2kb of the promoter, the reads in the ATAC-seq data were concentrated in the TSS region. **b** Repeatability between two replicates for ATAC-seq data. **c** Box plot showing the distribution of normalized counts for RNA-seq data. Each bar represents a sample. **d** Principal component analysis of $CRP^{stim}$ and control group based on expression of all genes. Each point is a sample, and the samples in the figure are colored by groups. $CRP^{stim}$, CRP stimulation.

Fig S7

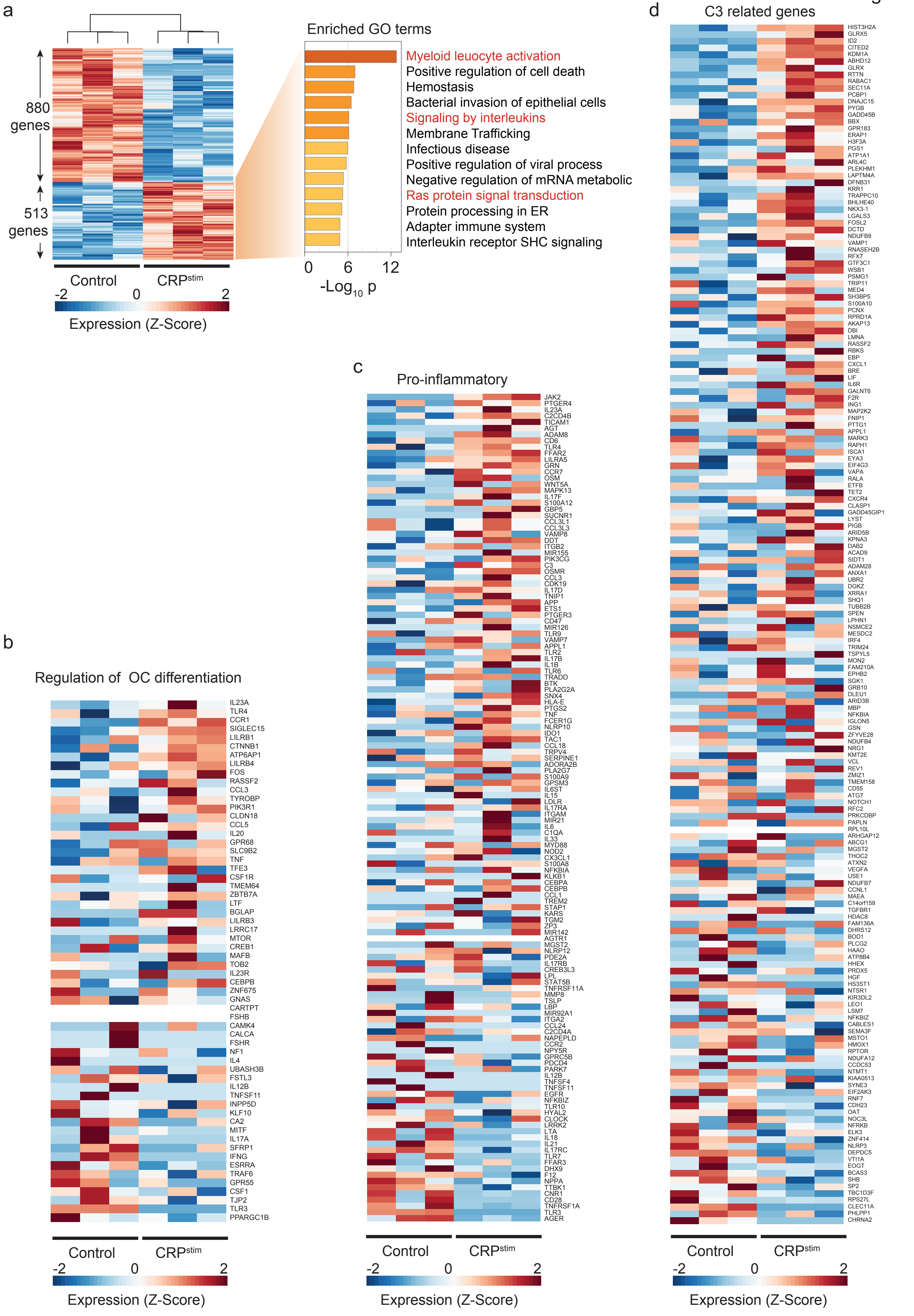
a
880 genes
513 genes
Control
CRP^stim
-2
0
2
Expression (Z-Score)
Enriched GO terms
Myeloid leucocyte activation
Positive regulation of cell death
Hemostasis
Bacterial invasion of epithelial cells
Signaling by interleukins
Membrane Trafficking
Infectious disease
Positive regulation of viral process
Negative regulation of mRNA metabolic
Ras protein signal transduction
Protein processing in ER
Adapter immune system
Interleukin receptor SHC signaling
0
6
12
-Log10 p
b
Regulation of OC differentiation
IL23A
TLR4
CCR1
SIGLEC15
LILRB1
CTNNB1
ATP6AP1
LILRB4
FOS
RASSF2
CCL3
TYROBP
PIK3R1
CLDN18
CCL5
IL20
GPR68
SLC9B2
TNF
TFE3
CSF1R
TMEM64
ZBTB7A
LTF
BGLAP
LILRB3
LRRC17
MTOR
CREB1
MAFB
TOB2
IL23R
CEBPB
ZNF675
GNAS
CARTPT
FSHB
CAMK4
CALCA
FSHR
NF1
IL4
UBASH3B
FSTL3
IL12B
TNFSF11
INPP5D
KLF10
CA2
MITF
IL17A
SFRP1
IFNG
ESRRA
TRAF6
GPR55
CSF1
TJP2
TLR3
PPARGC1B
Control
CRP^stim
-2
0
2
Expression (Z-Score)
c
Pro-inflammatory
JAK2
PTGER4
IL23A
C2CD4B
TICAM1
AGT
ADAM8
CD6
TLR4
FFAR2
LILRA5
GRN
CCR7
OSM
WNT5A
MAPK13
IL17F
S100A12
GBP5
SUCNR1
CCL3L1
CCL3L3
VAMP8
DDT
ITGB2
MIR155
PIK3CG
C3
OSMR
CCL3
CDK19
IL17D
TNIP1
APP
ETS1
PTGER3
CD47
MIR126
TLR9
VAMP7
APPL1
TLR2
IL17B
IL1B
TLR6
TRADD
BTK
PLA2G2A
SNX4
HLA-E
PTGS2
TNF
FCER1G
NLRP10
IDO1
TAC1
CCL18
TRPV4
SERPINE1
ADORA2B
PLA2G7
S100A9
GPSM3
IL6ST
IL15
LDLR
IL17RA
ITGAM
MIR21
IL6
C1QA
IL33
MYD88
NOD2
CX3CL1
S100A8
NFKBIA
KLKB1
CEBPA
CEBPB
CCL1
TREM2
STAP1
KARS
TGM2
ZP3
MIR142
AGTR1
MGST2
NLRP12
PDE2A
IL17RB
CREB3L3
LPL
STAT5B
TNFRSF11A
MMP8
TSLP
LBP
MIR92A1
ITGA2
CCL24
C2CD4A
NAPEPLD
CCR2
NPY5R
GPRC5B
PDCD4
PARK7
IL12B
TNFSF4
TNFSF11
EGFR
NFKBIZ
TLR10
HYAL2
CLOCK
LRRK2
LTA
IL18
IL21
IL17RC
TLR7
FFAR3
DHX9
F12
NPPA
TTBK1
CNR1
CD28
TNFRSF1A
TLR3
AGER
Control
CRP^stim
-2
0
2
Expression (Z-Score)
d
C3 related genes
HIST3H2A
GLRX5
ID2
CITED2
KDM1A
ABHD12
GLRX
RTTN
RABAC1
SEC11A
PCBP1
DNAJC15
PYGB
GADD45B
BBX
GPR183
ERAP1
H3F3A
PGS1
ATP1A1
ARL4C
PLEKHM1
LAPTM4A
DFNB31
KRR1
TRAPPC10
BHLHE40
NKX3-1
LGALS3
FOSL2
DCTD
NDUFB9
VAMP1
RNASEH2B
RFX7
GTF3C1
WSB1
PSMG1
TRIP11
MED4
SH3BP5
S100A10
PCNX
RPRD1A
AKAP13
DBI
LMNA
RASSF2
RBKS
EBP
CXCL1
BRE
LIF
IL6R
GALNT6
F2R
ING1
MAP2K2
FNIP1
PTTG1
APPL1
MARK3
RAPH1
ISCA1
EYA3
EIF4G3
VAPA
RALA
ETFB
TET2
CXCR4
CLASP1
GADD45GIP1
LYST
PIGB
ARID5B
KPNA3
DAB2
ACAD9
SIDT1
ADAM28
ANXA1
UBR2
DGKZ
XRRA1
SHQ1
TUBB2B
SPEN
LPHN1
NSMCE2
MESDC2
IRF4
TRIM24
TSPYL5
MON2
FAM210A
EPHB2
SGK1
GRB10
DLEU1
ARID3B
MBP
NFKBIA
IGLON5
GSN
ZFYVE28
NDUFB4
NRG1
KMT2E
VCL
REV1
ZMIZ1
TMEM158
CD55
ATG7
NOTCH1
RFC2
PRKCDBP
PAPLN
RPL10L
ARHGAP12
ABCG1
MGST2
THOC2
ATXN2
VEGFA
USE1
NDUFB7
CCNL1
MAEA
C14orf159
TGFBR1
HDAC8
FAM136A
DHRS12
BOD1
PLCG2
HAAO
ATP8B4
HHEX
PRDX5
HGF
HS3ST1
NTSR1
KIR3DL2
LEO1
LSM7
NFKBIZ
CABLES1
SEMA3F
MSTO1
HMOX1
RPTOR
NDUFA12
CCDC53
NTMT1
KIAA0513
SYNE3
EIF2AK3
RNF7
CDH23
OAT
NOC3L
NFRKB
ELK3
ZNF414
NLRP3
DEPDC5
VTI1A
EOGT
BCAS3
SHB
SP2
TBC1D3F
RPS27L
CLEC11A
PHLPP1
CHRNA2
Control
CRP^stim
-2
0
2
Expression (Z-Score)

**Fig. S7 CRP stimulation promotes OC-differentiation and pro-inflammation in OA-derived monocytes. a** Heatmap showing changes in gene expression in monocytes stimulated with CRP (10 μg/mL) for 12 hours (paired t-test $p < 0.05$ and fold change > 2). RNA-seq was performed for three independent biological replicates (monocytes derived from three different patients with OA). (right) Metascape was used to annotate genes; enriched GO terms after CRP stimulation. **b**, **c** Heatmaps show the expression levels of genes in the pro-inflammation (b) and regulation of OC-differentiation (c) gene sets. **d** Heatmap showing the expression levels of the genes of cluster 3 regions annotated by GREAT (identified in Fig. 2c). $CRP^{stim}$, CRP stimulation; OC, osteoclast; C3, cluster 3.

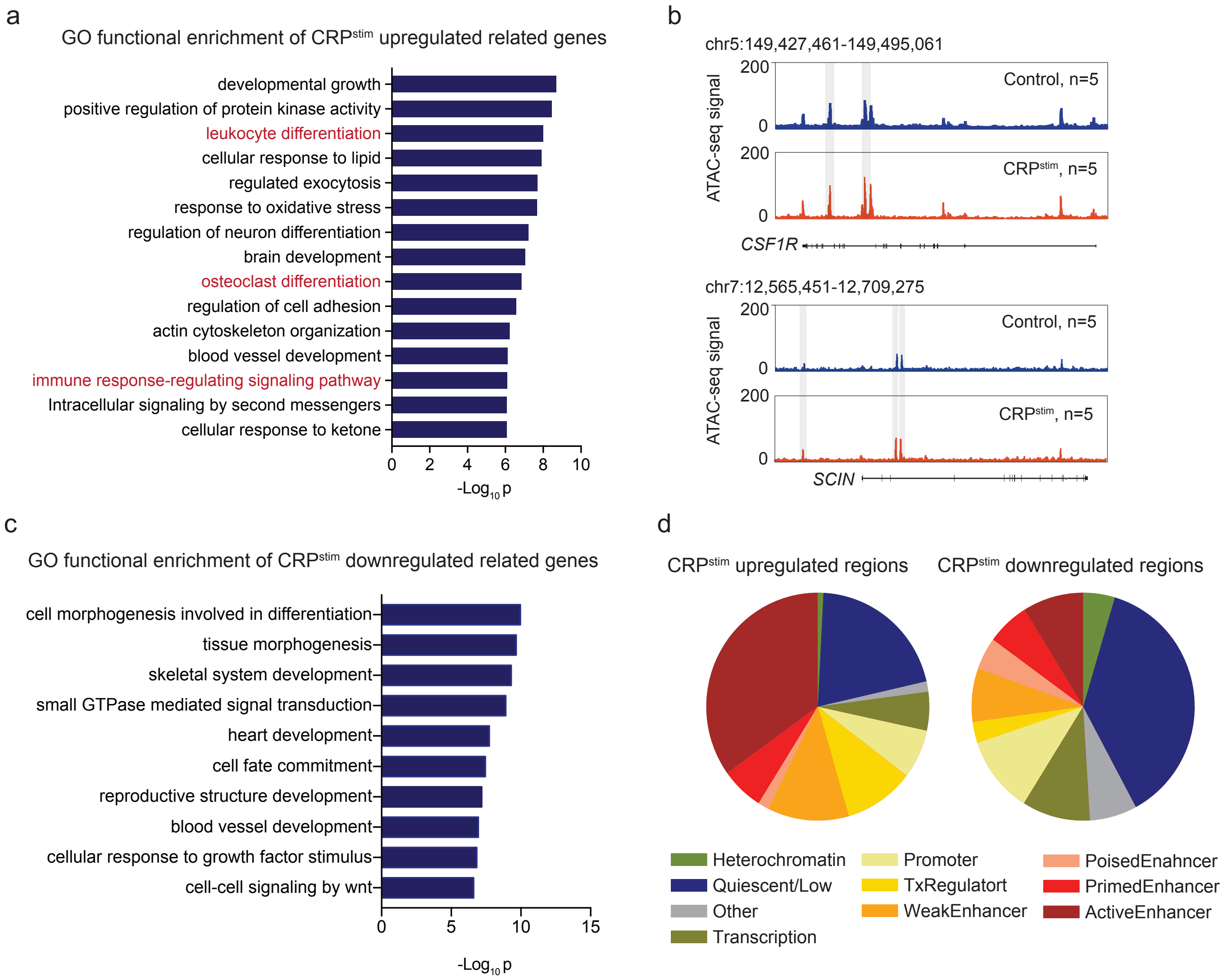


**Fig. S8 The functional genomic characteristics of differential regions after CRP stimulation. a** Enriched GO terms of genes related to the peaks upregulated after CRP stimulation by GREAT. **b** Normalized ATAC-seq profiles at the *CSF1R* and *SCIN* loci in the control and CRP$^{stim}$ groups. Shaded regions indicate peaks that are more accessible in CRP$^{stim}$. **c** Enriched GO terms among genes related to the peaks that became less accessible after CRP stimulation by GREAT. **d** Distribution of genomic features in the upregulated (left) and downregulated (right) regions after CRP stimulation. Different genomic features are annotated with different colours.

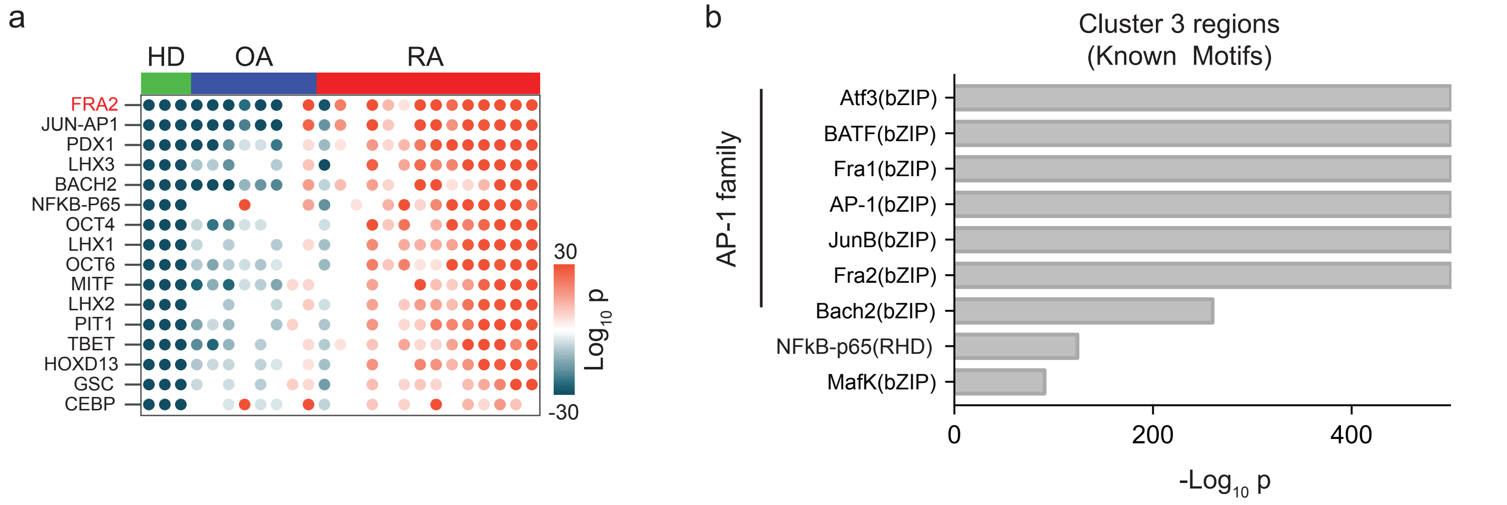


**Fig. S9 Transcription factor motifs enrich in cluster 3. a** Enrichment of transcription factor motifs in C3 for all samples. Each row is a motif, and each column is a sample. Values in the matrix indicate the significance of enrichment estimated by Genomica in terms of $-\text{Log}_{10}$ p. The top ranked motifs are shown. The colour bar indicates the category of each sample: HD, healthy donor; OA, osteoarthritis; RA, rheumatoid arthritis. **b** Transcription factor motifs enriched in the Cluster 3 peaks using HOMER and ranked by p value.
